# Metal–organic layer delivers 3-bromopyruvate to mitochondria for metabolic regulation and cancer radio-immunotherapy†

**DOI:** 10.1039/d4sc08563a

**Published:** 2025-02-17

**Authors:** Wangqing Bian, Xiaomin Jiang, Jinhong Li, Langston Tillman, Chaoyu Wang, Wenyao Zhen, Ralph R. Weichselbaum, Tobias Fromme, Wenbin Lin

**Affiliations:** a Department of Chemistry, The University of Chicago Chicago Illinois 60637 USA wenbinlin@uchicago.edu; b Chair of Molecular Nutritional Medicine, TUM School of Life Sciences, Technical University of Munich Freising Germany fromme@tum.de; c Department of Radiation and Cellular Oncology and Ludwig Center for Metastasis Research, The University of Chicago Chicago Illinois 60637 USA; d Pritzker School of Molecular Engineering, The University of Chicago Chicago Illinois 60637 USA; e EKFZ – Else Kröner Fresenius Center for Nutritional Medicine, Technical University of Munich Freising Germany

## Abstract

Abnormal cancer metabolism causes hypoxia and immunosuppression, limiting the anti-tumor efficacy of radiotherapy. Herein, we report a positively charged, mitochondria-targeted nanoscale metal–organic layer conjugated with 3-bromopyruvate (BP), BP/Hf_12_-Ir, for metabolic reprogramming and radiosensitization. BP/Hf_12_-Ir disrupts oxidative phosphorylation and glycolysis, reducing energy production and alleviating hypoxia to enhance radiotherapy and anti-tumor immunity. BP/Hf_12_-Ir in combination with X-ray irradiation inhibits tumor growth by 95% and prevents lung metastasis in mouse models. When further combined with immune checkpoint blockade, this treatment induces robust anti-tumor immunity, achieving 98% tumor growth inhibition.

## Abstract 2

We design a mitochondria-targeted, BP-loaded nanoscale MOL for metabolic reprogramming and radiosensitization, disrupting energy production and hypoxia to suppress tumor growth and prevent metastasis by enhancing radiotherapy and antitumor immunity.

## Graphical abstract

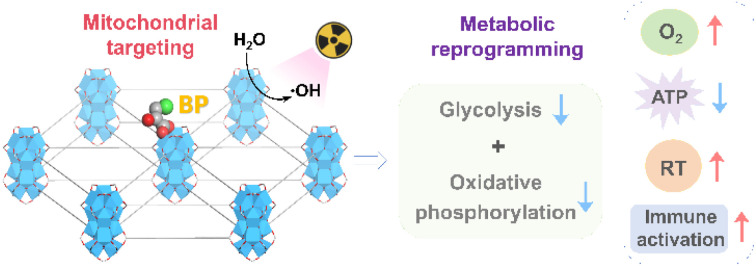

## Introduction

Cancer cells undergo metabolic reprogramming to support their proliferation through increased glycolysis.^1–4^ Targeting altered metabolic pathways can disrupt energy production and biosynthesis processes to induce cancer cell death.^4–8^ However, when glycolysis is inhibited, cancer cells can switch to mitochondrial oxidative phosphorylation (OXPHOS) for energy production.^3,9–11^ Simultaneous inhibition of glycolysis and mitochondrial metabolism overcomes the compensatory mechanisms of cancer cells to effectively disrupt energy production and kill cancer cells.

Hexokinase II (HK-II) catalyzes the first step in glycolysis by converting glucose to glucose-6-phosphate^12,13^ and presents a prime target for disrupting cancer cell metabolism.^14–16^ HK-II attaches to the outer membrane of mitochondria (OMM) through interaction with the voltage-dependent anion channel (VDAC).^12,17^ ATP from OXPHOS fuels mitochondrion-bound HK-II to drive glucose-6-phosphate synthesis.^12,18–20^ Inhibition of HK-II can interfere with its binding to VDAC, triggering apoptosis in cancer cells.^12,13^ As an analog of pyruvate and lactate, 3-bromopyruvate (BP) inhibits HK-II through covalent modification of cysteine residues to decrease glycolysis. HK-II inhibition also reduces oxygen consumption to alleviate tumor hypoxia and sensitize cancer cells to radiotherapy.^17,21–23^

Radiotherapy is an important treatment for the majority of cancer patients.^24–28^ Radiotherapy induces cancer cell death by directly damaging DNAs or indirectly decomposing vital biomolecules *via* generating reactive oxygen species (ROS). However, radiotherapy is only effective at high X-ray doses, which cause severe side effects in cancer patients.^29–31^ Significant efforts have been devoted to developing nanotherapeutics to improve cancer treatments including radiotherapy.^32–36^ We have recently demonstrated radioenhancement effects of high-Z element nanoscale metal–organic layers (MOLs), a monolayer version of metal–organic frameworks.^37–39^ Ultrathin MOLs also facilitates ROS diffusion to increase its cytotoxicity to tumor cells,^40^ and can be modified with functional molecules to synergize with MOL-mediated radioenhancement.^41–44^

Recently, Fu *et al.* loaded BP into Hf-TCPP nanoscale metal–organic framework to overcome RT resistance.^45^ Shen *et al.* co-loaded BP and metformin into ZIF-90 to alter metabolic regulation, which increased the effectiveness of redox-based anticancer therapy.^46^ Meng *et al.* co-loaded BP and glucose oxidase into ZIF-8 to disrupt redox balance in a hepatocellular carcinoma cell line and achieve an anti-tumor effect.^47^ However, the synergistic inhibition of glycolysis and OXPHOS in combination with RT for antitumor treatment remains unexplored and BP has not been coordinated to MOLs for RT enhancement.

**Fig. 1:**
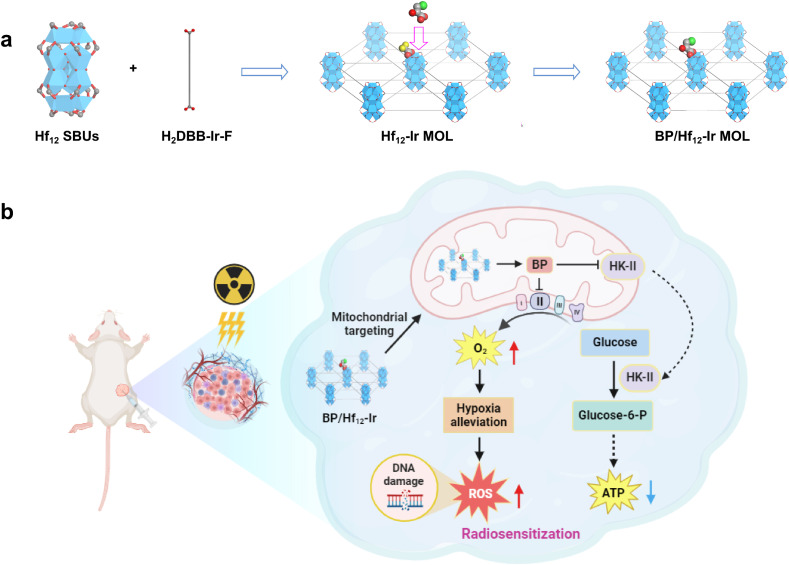
(a) Synthesis of BP/Hf_12_-Ir MOL. (b) BP/Hf_12_-Ir-mediated metabolic reprogramming enhances mitochondria-targeted radiotherapy.

Herein, we report a bifunctional MOL, BP/Hf_12_-Ir, with BP conjugated to the Hf_12_-Ir MOL comprising Hf_12_ secondary building units (SBUs) and Ir(DBB)[dF(CF_3_)ppy]_2_^+^ (DBB = 4,4′-di(4-benzoato)-2,2′-bipyridine), [dF(CF_3_)ppy = 2-(2,4-difluorophenyl)-5-(trifluoromethyl)pyridine] ligands for simultaneous mitochondrial metabolic regulation and radioenhancement (Fig. 1). The positively charged MOL targets mitochondria, where high intracellular phosphate concentrations trigger BP release from BP/Hf_12_-Ir, enhancing RT by alleviating hypoxia through the inhibition of mitochondrial function and glycolytic metabolism.^48–52^ Consequently, BP/Hf_12_-Ir in combination with X-ray irradiation potently regresses colorectal carcinoma and breast cancer in mouse models.

## Results and discussion

### Synthesis and characterization of Hf_12_-Ir MOL and BP/Hf_12_-Ir

**Fig. 2:**
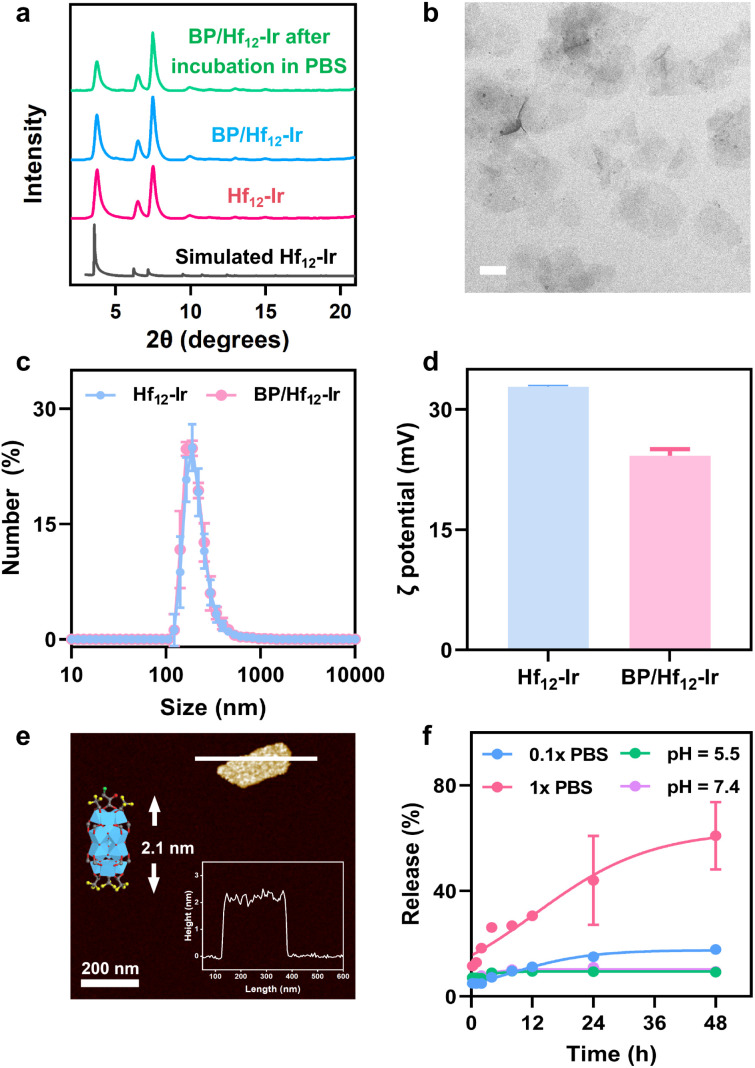
(a) PXRD patterns of Hf_12_-Ir and BP/Hf_12_-Ir before and after soaking in PBS for 24 h. (b) Number-averaged diameters of Hf_12_-Ir and BP/Hf_12_-Ir in water. (c) TEM image of BP/Hf_12_-Ir. Scale bar = 200 nm. (d) *ζ* potentials of Hf_12_-Ir and BP/Hf_12_-Ir in water. (e) AFM topographic image, height profile (inset, right), and modeled height (inset, left) of BP/Hf_12_-Ir. (f) Release profiles of BP from BP/Hf_12_-Ir.

Hf_12_-Ir MOL was synthesized through a solvothermal reaction between HfCl_4_ and H_2_DBB-Ir-F in *N*,*N*-dimethylformamide at 80 °C with trifluoroacetic acid (TFA) as a modulator (Fig. S1–S4†). Powder X-ray diffraction (PXRD) and ^1^H NMR studies established Hf_12_-Ir as a 2D network of Hf_12_ SBUs bridged by DBB-Ir-F ligands with a formula of Hf_12_(μ_3_-O)_8_(μ_3_-OH)_8_(μ_2_-OH)_6_(DBB-Ir-F)_6_(TFA)_6_ (Fig. 2a and S5†). Transmission electron microscopy (TEM) and atomic force microscopy (AFM) imaging confirmed the monolayer morphology with a diameter of ∼190 nm and a thickness of ∼1.9 nm (Fig. S6†). Dynamic light scattering (DLS) studies gave a size of 170.6 ± 1.7 nm Hf_12_-Ir (Fig. 2c).

BP/Hf_12_-Ir was synthesized by treating Hf_12_-Ir with BP at room temperature through replacing TFA capping agents with BP *via* carboxylate exchange. PXRD, TEM, and DLS studies showed that BP/Hf_12_-Ir retained the crystallinity and size of Hf_12_-Ir (Fig. 2a–c). ^1^H NMR studies indicated partial substitution of TFA by BP, yielding BP/Hf_12_-Ir with the formula of Hf_12_(μ_3_-O)_8_(μ_3_-OH)_8_(μ_2_-OH)_6_(DBB-Ir-F)_6_(TFA)_1.6_(BP)_4.4_ (Fig. S7–S10†). The *ζ* potential of BP/Hf_12_-Ir remained positive at 24.2 mV, which endows its mitochondrial targeting ability (Fig. 2d). The thickness of BP/Hf_12_-Ir slightly increased to 2.1 nm, due to the capping of Hf_12_ SBUs by larger BP groups (Fig. 2e). Furthermore, BP/Hf_12_-Ir remained stable and retained its crystallinity after incubation in phosphate-buffered saline (PBS) buffer for 24 hours (Fig. 2a). Liquid chromatography-mass spectrometry (LC-MS) analysis showed that incubation of BP/Hf_12_-Ir in 0.1× PBS and 1× PBS released 17.9% and 60.4% BP, respectively, in 48 hours (Fig. 2f). Less than 10% BP was released in pH 5.5 and 7.4 aqueous solutions. Thus, high phosphate concentrations inside cells can trigger the release of BP from BP/Hf_12_-Ir.

### *In vitro* study of BP/Hf_12_-Ir

**Fig. 3:**
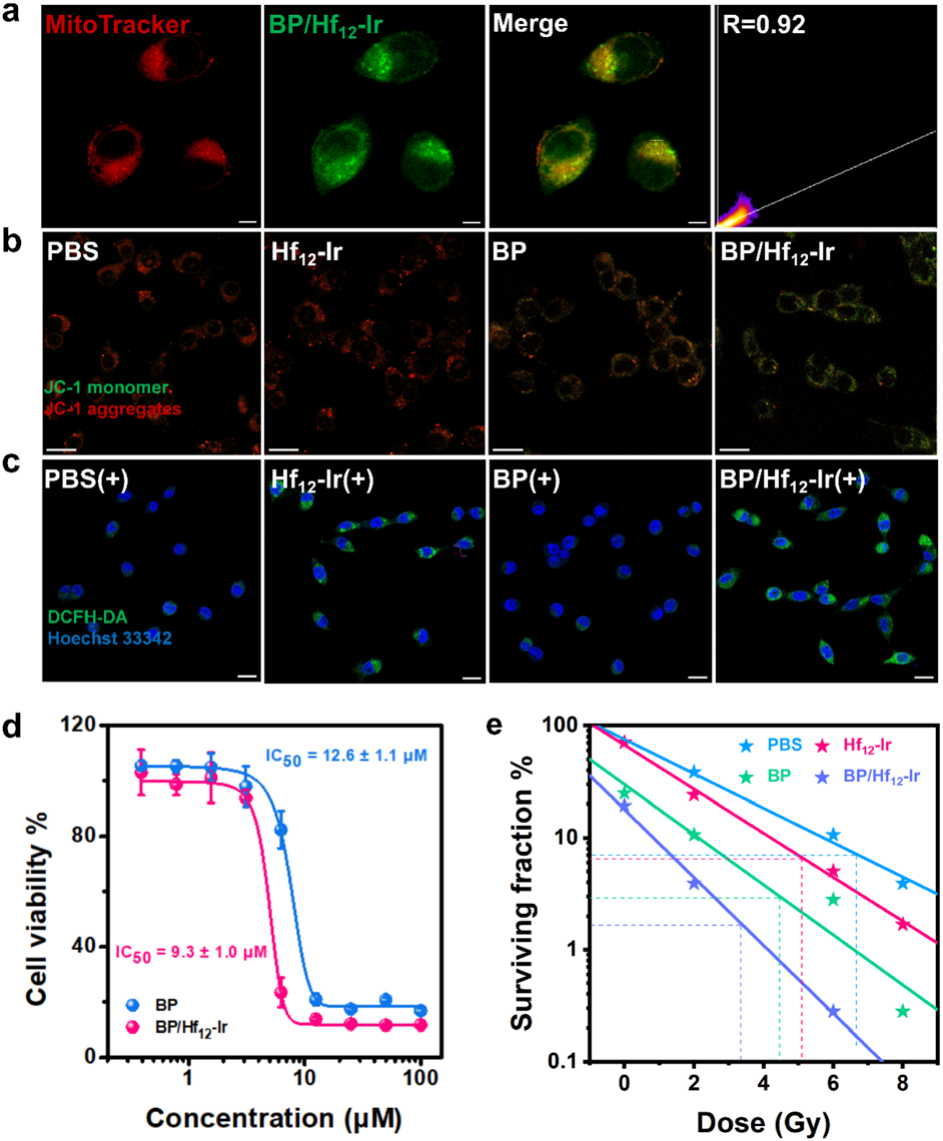
(a) CLSM images and colocalization of BP/Hf_12_-Ir with mitochondria. (b) Mitochondrial potential depolarization by JC-1 assay. (c) DCFH-DA assay showing total ROS (green) generation. (d) Cell viability assay of BP and BP/Hf_12_-Ir. (e) Clonogenic assay after different treatments. CT26 cells were used in all studies. Scale bar = 20 μm in a–c. *n* = 3 in d and e.

BP/Hf_12_-Ir showed time-dependent uptake in CT26 murine colon cancer cells (Fig. S11†). Mitochondrial targeting ability of BP/Hf_12_-Ir was assessed by co-localization of Mito-Tracker and Hf_12_-Ir luminescence by confocal laser scanning microscopy (CLSM). BP/Hf_12_-Ir was abundantly present in mitochondria with a co-localization coefficient (Pearson's *R* value) of 0.92 (Fig. 3a and S12†).

As mitochondria are cellular ATP factories with high oxygen concentrations, mitochondria-targeted Hf_12_-Ir is expected to efficiently generate ROS *via* radiosensitization. CLSM imaging with 2′,7′-dichlorodihydrofluorescein diacetate (DCFH-DA) probe revealed that Hf_12_-Ir plus 6 Gy X-ray [denoted Hf_12_-Ir(+)] and BP/Hf_12_-Ir(+) exhibited stronger total ROS signals than PBS(+) in CT26 cells (Fig. 3c, S14 and 15†). Western blot studies showed that Hf_12_-Ir(+) increased phosphorylated histone H2A.X (γ-H2AX) levels over PBS(+) in CT26 cells, indicating more DNA double-strand breaks (DSBs) (Fig. S16†). The long-term proliferation of CT26 cells under different X-ray doses was assessed by clonogenic assays. Compared to PBS(+), Hf_12_-Ir(+) and BP/Hf_12_-Ir(+) showed similar radiation enhancement factors at 10% survival rates (REF_10_) of 1.28 and 1.36 over PBS and BP, respectively (Fig. 3e and S17†). These results show that mitochondria-targeted Hf_12_-Ir provides potent radiosensitization to damage DNAs and kill cancer cells.

The cytotoxicity of BP and BP/Hf_12_-Ir was assessed in CT26 cells by MTS assay. While Hf_12_-Ir showed no cytotoxicity (Fig. S13†), BP and BP/Hf_12_-Ir showed high toxicity with IC_50_ values of 12.6 ± 1.1 μM and 9.3 ± 1.0 μM, respectively (Fig. 3d). JC-1 staining was performed to investigate the effect of BP on the mitochondrial membrane potential (MMP) which plays a key role in OXPHOS for ATP synthesis. Hf_12_-Ir did not influence the MMP of CT26 cells, as evidenced by the unchanged J-aggregate (red) and J-monomer (green) signals (Fig. 3b). In contrast, BP caused significantly decreased J-aggregate signals and increased J-monomer signals in CT26 cells. BP/Hf_12_-Ir treatment further increased J-monomer signals over BP. MMP depolarization by BP/Hf_12_-Ir also induced strong apoptosis (Fig. S18†). BP/Hf_12_-Ir increased the percentages of apoptotic cells to 43.70% from 1.79% for PBS and 5.90% for BP.

### Metabolic reprogramming by BP/Hf_12_-Ir

**Fig. 4:**
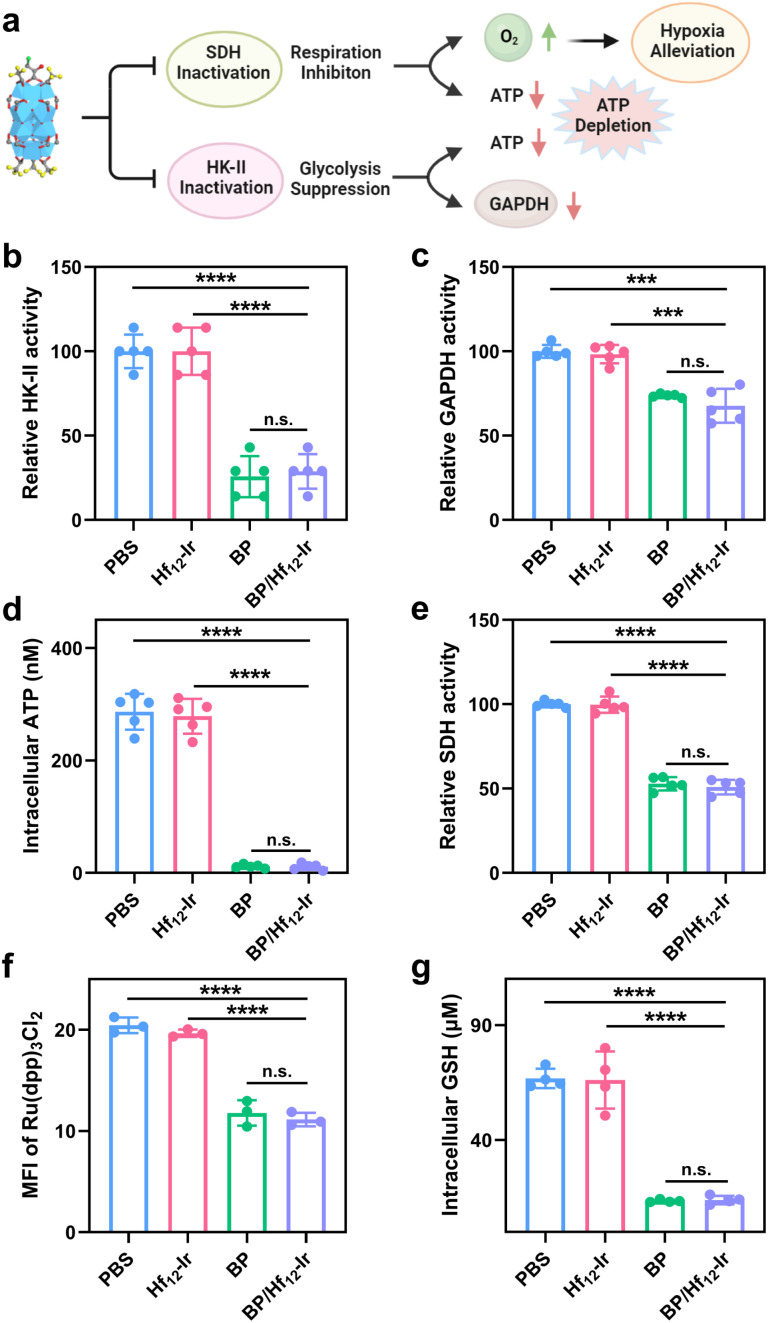
(a) Schematic illustration of mitochondrial and glycolysis metabolic reprogramming by BP/Hf_12_-Ir. (b) HK-II activities, (c) GAPDH activities, (d) Intracellular ATP levels, (e) SDH activities, (f) hypoxia-indicating Ru(dpp)_3_Cl_2_ luminescence signals, and (g) intracellular GHS concentrations after different treatments. *n* = 3, ***p < 0.001; ****p < 0.0001.

We next studied the disruption of glycolysis and mitochondrial respiration by assessing key protein expressions, ATP and GSH levels, and mitochondrial O_2_ levels (Fig. 4a). BP and BP/Hf_12_-Ir decreased HK-II activity in CT26 cells by 25.8% and 28.8%, respectively, from PBS (Fig. 4b). As mitochondrial HK-II is key for glycolysis, BP and BP/Hf_12_-Ir reduced downstream GAPDH activity by 73.8% and 67.7%, respectively, from PBS (Fig. 4c). BP and BP/Hf_12_-Ir also significantly reduced intracellular ATP concentration to 11.6 μM and 11.7 μM, respectively, from 286.3 μM for PBS (Fig. 4d).

BP can also disrupt the mitochondrial tricarboxylic acid cycle and OXPHOS by inhibiting succinate dehydrogenase (SDH) activity to reduce oxygen consumption (Fig. 4a). BP and BP/Hf_12_-Ir decreased SDH activity by 52.9% and 50.9%, respectively, from PBS (Fig. 4e). We used Ru(dpp)_3_Cl_2_ to assess mitochondrial oxygen levels by CLSM. Incubation of CT26 cells under hypoxic conditions (0.5% O_2_) led to strong red luminescence from Ru(dpp)_3_Cl_2_ for PBS and Hf_12_-Ir groups due to O_2_ depletion. BP and BP/Hf_12_-Ir reduced Ru(dpp)_3_Cl_2_ luminescence by 57.7% and 54.4%, respectively (Fig. 4f and S19†). Moreover, BP and BP/Hf_12_-Ir reduced intracellular GSH concentration to 13.5 μM and 13.9 μM, respectively, from 66.7 μM for PBS (Fig. 4g). These results indicate hypoxia alleviation by BP and BP/Hf_12_-Ir to increase intracellular O_2_ levels, which can enhance the efficacy of RT. Hf_12_-Ir did not change HK-II, GAPDH, and SDH activities, ATP production, and mitochondrial O_2_ level and intracellular GSH concentration from PBS, further supporting the inhibition of glycolysis and mitochondrial respiration by the released BP.

### *In vivo* study of BP/Hf_12_-Ir

**Fig. 5:**
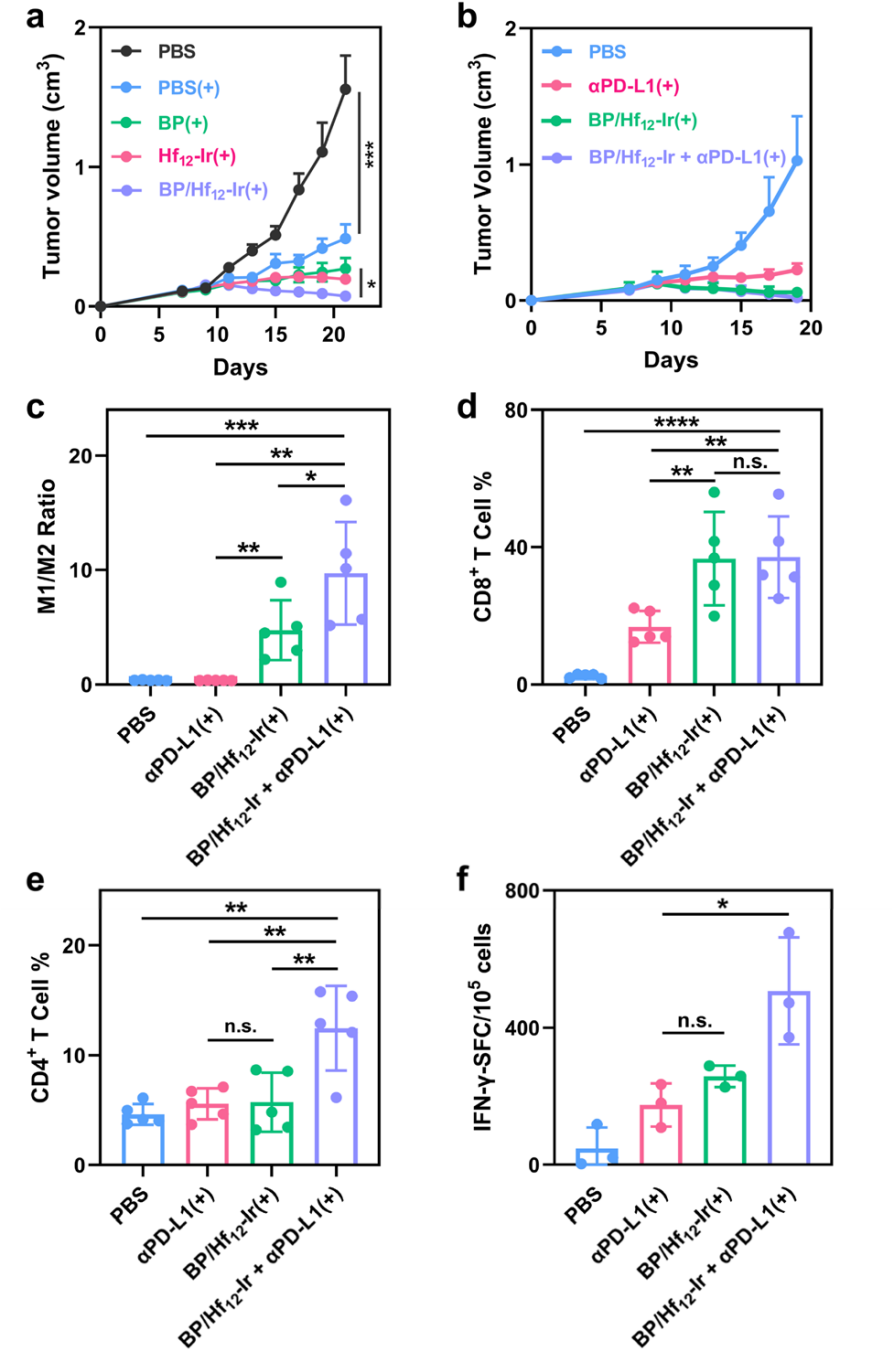
(a and b) Growth curves of CT26 tumors (a, *n* = 5) and 4T1 tumors (b, *n* = 5) after different treatments. (c–e) M1/M2 macrophage ratios (c), CD8^+^ T cell (d), and CD4^+^ T cell (e) subpopulations in CT26 tumors. (f) ELISpot assay detecting SPSYVYHQF antigen-specific IFN-γ secreting splenocytes (*n* = 3).

The antitumor efficacy of BP/Hf_12_-Ir(+) was evaluated in subcutaneous CT26 and 4T1 tumor models. Mice with established CT26 tumors (∼100 mm^3^) were intratumorally injected with PBS, Hf_12_-Ir, BP, or BP/Hf_12_-Ir (0.5 μmol Hf_12_-Ir or/and 0.2 μmol BP) on days 7 and 9 post tumor inoculation (Fig. S22†). The tumors were irradiated with 2 Gy X-ray for 6 daily fractions. While Hf_12_-Ir(+) and BP(+) significantly slowed tumor growth with tumor growth inhibition (TGI) values of 87.3% and 82.6%, respectively, BP/Hf_12_-Ir(+) synergized the effects of RT and metabolic reprogramming to provide a TGI of 95.2% (Fig. 5a).

The metabolic stress induced by BP can lead to immunogenic cell death (ICD) and the release of damage-associated molecular patterns (DAMPs) from cancer cells. BP/Hf_12_-Ir(+) treatment showed significant ATP release (Fig. S20†) and surface translocation of calreticulin (CRT) (Fig. S21†), both hallmarks of ICD. These DAMPs play a crucial role in stimulating the immune system to activate the tumor microenvironment (TME). As BP/Hf_12_-Ir(+) doubled PD-L1 expression over PBS in CT26 cells (Fig. S24†), we tested its combination with immune checkpoint blockade to further enhance antitumor effects *via* T cell reinvigoration. BP/Hf_12_-Ir(+) plus αPD-L1 (100 μg per mouse) significantly enhanced the anti-tumor efficacy to regress CT26 and 4T1 tumors with TGI values of 98.2% and 97.9%, respectively (Fig. 5b and S23a†), and eradicate tumors in 60% of the mice in both tumor models. In comparison, αPD-L1(+) showed TGI values of 70.6% and 78.1% for CT26 and 4T1 tumors, respectively, while BP/Hf_12_-Ir(+) afforded TGI values of 95.2% and 91.6% for CT26 and 4T1 tumors, respectively.

The anti-metastatic effect of BP/Hf_12_-Ir(+) was evaluated on an orthotopic 4T1 model, which is known to develop lung metastasis. Histological analysis of lung tissues by hematoxylin and eosin (H&E) staining revealed that BP/Hf_12_-Ir(+) and BP/Hf_12_-Ir + αPD-L1(+) groups showed strong anti-metastatic effects with 1.6% and 0% metastatic rates, respectively, while PBS and αPD-L1(+) groups showed metastatic rates of 6.4% and 26.0%, respectively (Fig. S31 and Table S1†).

To investigate the tumor immune microenvironment, we profiled leukocytes in tumors 7 days after the last X-ray irradiation by flow cytometry. BP/Hf_12_-Ir(+) and BP/Hf_12_-Ir + αPD-L1(+) induced significant polarization of macrophages to the pro-inflammatory M1-like state, leading to 13.2- and 27.0-fold higher M1/M2 ratios, respectively, over PBS (Fig. 5c). BP/Hf_12_-Ir(+) and BP/Hf_12_-Ir + αPD-L1(+) groups significantly induced cytotoxic CD8^+^ T cell infiltration into the tumors (Fig. 5d, S28 and 29†). Additionally, BP/Hf_12_-Ir + αPD-L1(+) enhanced the helper (CD4^+^) T cell population in the TME by 2.7-fold over PBS (Fig. 5e and S28†). These results show that BP/Hf_12_-Ir + αPD-L1(+) exhibits superior anti-tumor effects by activating both innate and adaptive immune responses.

An IFN-γ enzyme-linked immunospot (ELISpot) assay was performed on splenocytes from treated CT26 tumor-bearing mice. BP/Hf_12_-Ir(+) and BP/Hf_12_-Ir + αPD-L1(+) showed 5.5- and 10.8-fold more spot-forming cells (SFC) than PBS (Fig. 5f and S32†), indicating antigen-specific antitumor effects and systemic antitumor immunity from these treatments.

We sectioned CT26 tumors for H&E, terminal deoxynucleotidyl transferase dUTP nick end labeling (TUNEL), γ-H2AX, Ki67 and carbonic anhydrase 9 (CA9) staining. BP/Hf_12_-Ir(+)-treated tumors showed the lowest cancer cell densities with pervasive nuclear chromatin pyknosis and cytoplasm disappearance, the highest levels of DNA fragmentations, and the lowest levels of cell proliferation (Fig. S26†). Additionally, BP(+)- and BP/Hf_12_-Ir(+)-treated tumors displayed decreased levels of CA9, suggesting hypoxia alleviation by BP in the tumors (Fig. S27†). Lastly, the mice in all treatment groups showed steady body weights (Fig. S23 and S30†) and normal histologies of major organs (Fig. S25†), highlighting the lack of general toxicity for BP/Hf_12_-Ir(+).

## Conclusions

In this study, we developed a positively charged, mitochondria-targeted, and BP-conjugated MOL for metabolic reprogramming and radiosensitization. BP/Hf_12_-Ir inhibits oxidative phosphorylation and glycolysis, reducing energy production and alleviating hypoxia to enhance radiotherapy and anti-tumor immunity. BP/Hf_12_-Ir-mediated radiotherapy inhibits tumor growth by 95% and prevents lung metastasis. When combined with immune checkpoint blockade, the treatment potently regresses the tumors with 98% tumor growth inhibition by inducing robust anti-tumor immunity. This work uncovers an innovative approach to enhance radiotherapy efficacy and strengthen anti-tumor immune responses.

## Data availability

The datasets supporting this article have been uploaded as part of the ESI.†

## Author contributions

W. Bian, X. Jiang, W. Lin and T. Fromme conceived the project and wrote the manuscript. W. Bian and J. Li synthesized the material and characterized the material. W. Bian, T. Langston C. Wang and W. Zhen conducted the *in vitro* and *in vivo* experiments.

## Conflicts of interest

There are no conflicts to declare.

## Supplementary Material

SC-016-D4SC08563A-s001
