# Supplementary material for: Metal–organic layer delivers 3-bromopyruvate to mitochondria for metabolic regulation and cancer radio-immunotherapy
Source: Chem Sci. 2025 Feb 17;16(12):5234–40. doi: 10.1039/d4sc08563a (PMC11843481; doi:10.1039/d4sc08563a)
Supplement: SC-016-D4SC08563A-s001 [file SC-016-D4SC08563A-s001.pdf]

Supplementary Information (SI) for Chemical Science.
This journal is © The Royal Society of Chemistry 2025

Supporting Information

Metal-Organic Layer Delivers 3-Bromopyruvate to Mitochondria for Metabolic Regulation and Cancer Radio-Immunotherapy

Wangqing Bian,[†,a,b] Xiaomin Jiang,[†,a,c] Jinhong Li,[a] Langston Tillman,[d] Chaoyu Wang,[a,c] Wenyao Zhen,[a,c] Ralph R. Weichselbaum,[c] Tobias Fromme*,[b,e] and Wenbin Lin*,[a,c]

[a]Department of Chemistry, The University of Chicago, Chicago, Illinois 60637, United States

[b]Chair of Molecular Nutritional Medicine, TUM School of Life Sciences, Technical University of Munich, Freising, Germany

[c]Department of Radiation and Cellular Oncology and Ludwig Center for Metastasis Research, The University of Chicago, Chicago, Illinois 60637, United States

[d]Pritzker School of Molecular Engineering, The University of Chicago, Chicago, Illinois 60637, United States

[e]EKFZ - Else Kröner Fresenius Center for Nutritional Medicine, Technical University of Munich, Freising, Germany

[†]These authors contributed equally.

E-mail: wenbinlin@uchicago.edu; fromme@tum.de

Table of contents

1. Experimental Section 2
2. Synthesis and Characterization of Hf_{12}-Ir MOL and BP/Hf_{12}-Ir MOL 3
3. Characterization, *In Vitro* Study, and *In Vivo* Study of BP/Hf_{12}-Ir MOL. 4
4. Supplementary Figures 12
5. References 31

1. Experimental Section

1.1. Materials, Cell lines, and Animals

3-Bromopyruvate (BP) and all starting chemicals for the synthesis of nanoscale metal-organic layers (MOLs) were purchased from Sigma-Aldrich (USA) or Fisher Scientific (USA) unless otherwise stated and used directly without further purification. Phosphate buffered saline (PBS) was purchased from Corning (USA). 2',7'-Dichlorodihydrofluorescein diacetate (DCFH-DA) was purchased from Invitrogen.

Murine colon carcinoma cell line CT26 and murine mammary carcinoma cell line 4T1 were purchased from American Type Culture Collection (Rockville, USA), and cultured in RPMI-1640 medium with fetal bovine serum (FBS, 10%), penicillin G sodium (100 U/mL) and streptomycin sulfate (100 μg/mL). All the cells were maintained in a humidified atmosphere with 5% CO_2 at 37 °C.

BALB/c female mice (6 weeks, 18-22 g) were purchased from Charles River Laboratories Inc. (Wilmington, MA, USA). The experiments were conducted with the approval of Institutional Animal Care and Use Committee (IACUC) at the University of Chicago (PHS Assurance #D16-00322 (A3523-01)).

1.2. Instruments

^{1}H NMR spectra were recorded on a 500 MHz Bruker Avance-III spectrometer at 500 MHz and referenced to the proton resonance resulting from incomplete deuteration of DMSO-*d6* ($\delta = 2.50$). UV-Vis spectra were collected using a Shimadzu UV-2600 UV-Vis spectrophotometer. Dynamic light scattering (DLS) measurements were performed on a Malvern Zetasizer Nano ZS instrument. Transmission electron microscopy (TEM) was carried out on a TECNAI Spirit. Atomic force microscopy (AFM) images were taken on a Bruker Multimode 8-HR instrument. Powder X-ray diffraction (PXRD) data was collected at room temperature on a SAXSLAB's GANESHA using a Cu Kα radiation source (λ = 1.54178 Å). Inductively coupled plasma-mass spectrometry (ICP-MS) data were obtained with an Agilent 7700x ICP-MS and analyzed using ICP-MS Mass Hunter version B01.03. Confocal laser scanning microscope (CLSM) images were collected on a Leica SP8 laser scanning confocal microscope at the Integrated Light Microscopy Facility at the University of Chicago, and analysis was performed with Image J software (NIH, USA). Flow cytometry data were collected on an LSR-Fortessa-4-15 and a CYTEK AURORA, and subsequently analyzed using FlowJo software (Tree Star, USA). For test tubes and in vitro X-ray irradiation,

an RT250 orthovoltage X-ray machine (Philips, USA) was used with a fixed setting of 250 kVp, 15 mA, and a built-in 1 mm Cu filter. For animal irradiation, an X-RAD 225 image-guided biological irradiator (Precision X-ray Inc., USA) was used with a voltage of 225 kVp, current of 13 mA, a 0.3 mm Cu filter, and a 15 mm collimator. The X-ray dose rate of X-RAD 225 was 0.04167 Gy/second. The X-ray dosimetry of both instruments was regularly calibrated with an ionization chamber by the Department of Radiation Oncology at the University of Chicago. The histological slides were scanned on an Olympus VS200 Scan 40x whole slide scanner by Integrated Light Microscopy Core and analyzed with the QuPath-0.2.3 software. The absorbance and fluorescence from well plates were read by a BioTek Synergy HTX microplate reader.

1.3. Statistical analysis

P values were calculated using Microsoft Excel software with Student's two-tailed t-test. Statistical significance is indicated in all figures as follows: *, $p<0.05$, **, $p<0.01$, ***, $p<0.001$, and ****, $p<0.0001$.

2. Synthesis and Characterization of Hf_{12}-Ir MOL and BP/Hf_{12}-Ir MOL

2.1. Synthesis of H_2DBB-Ir-F

Ir(DBB)[dF(CF_3)ppy$]_2^+$ [H_2DBB-Ir-F, DBB = 4,4'-di(4-benzoato)-2,2'- bipyridine; dF(CF_3)ppy = 2-(2,4-difluorophenyl)-5-(trifluoromethyl)pyridine] was synthesized as shown in Figure S1 according to the literature report.[1]

2.2. Synthesis of Hf_{12}-Ir MOL

Hf_{12}-Ir MOL was synthesized as shown in Figure S4 according to a literature report.[1] To a 4 mL glass vial was added 0.5 mL of $HfCl_4$ solution [2.0 mg/mL in N,N-dimethylformamide (DMF)], 0.5 mL of H_2DBB-Ir-F solution (4.0 mg/mL in DMF), 2 μL of trifluoroacetic acid (TFA), and 5 μL of water. The reaction mixture was kept in an 80 °C oven for 24 hours. The yellow precipitate was collected by centrifugation and washed with DMF and ethanol. The yield of Hf_{12}-Ir MOL was 58%.

2.3. Synthesis of BP/Hf_{12}-Ir MOL

To a 20 mL glass vial was added 6.59 mL of Hf_{12}-Ir MOL dispersion (3.34 mM in EtOH based on Hf), 0.66 mL of BP solution (100 mM in EtOH) and a stirring bar. The mixture was stirred at room temperature in the dark overnight. BP/Hf_{12}-Ir was collected by centrifugation and washed with ethanol three times. The

yield of BP/Hf_{12}-Ir was 90%.

To determine BP loading in BP/Hf_{12}-Ir, 2 mL of BP/Hf_{12}-Ir dispersion was dried under vacuum. To the resultant solid was added 540 µL DMSO-*d6*, 30 µL D_3PO_4 and 30 µL D_2O. The mixture was sonicated for 30 min and then analyzed by ^{1}H NMR. The BP loading was calculated as follows:

$$(\%) = [\frac{n(3BrPA)3.47+n(3BrPA)4.54}{n(DBB)}] \times 100\%$$

3. Characterization, *In Vitro* Study, and *In Vivo* Study of BP/Hf_{12}-Ir

3.1. BP Release Profiles

BP/Hf_{12}-Ir was freshly prepared and redispersed in 1× PBS or 0.1× PBS (200 µL/tube) in 1.5 mL Eppendorf tubes (n = 3). The EP tubes were transferred to a 37 °C incubator. The supernatants (100 µL/tube) were collected at 0 h, 0.25 h, 1 h, 2 h, 4 h, 8 h, 12 h, 24 h and 48 h by centrifugation at 14,000 rpm. The supernatants were collected and derivatized as previously reported.[2] The resulting derivatized supernatants were directly subjected to LC-MS analysis to determine the BP contents.

3.2. DLS and Zeta-Potential Measurement

Dynamic light scattering (DLS) and zeta-potential measurements were performed on BP/Hf_{12}-Ir and Hf_{12}-Ir MOLs. 1 mL of BP/Hf_{12}-Ir or Hf_{12}-Ir aqueous dispersion at a DBB concentration of 5 µM was added to the cell for DLS and ζ potential measurements.

3.3. Cellular uptake

The cellular uptake of BP/Hf_{12}-Ir was evaluated in CT26 cells. The cells were seeded in 6-well plates (density: 2×10^5 cells/well) and cultured in RPMI-1640 medium containing 10% FBS overnight. BP/Hf_{12}-Ir was added to each well at an equivalent DBB concentration of 5 µM and incubated for 0, 2, 4, 6, and 8 h. The medium was removed, and cells were washed twice with PBS, trypsinized, counted, and then digested with 99% HNO_3 and 1% HF (990 µL HNO_3, 10 µL HF) for 48 hours. The cellular uptake of BP/Hf_{12}-Ir was determined by measuring the Hf content using ICP-MS.

3.4. Mitochondria Targeting

CT26 cells (2.0×10^5 cells) were seeded in 35 mm Petri with 2 mL of RPMI-1640 medium and cultured overnight. BP/Hf_{12}-Ir at a Hf concentration of 125 µM was added to each well and further incubated for 4

h. The cells were then washed once with cold DPBS and exchanged with warm medium containing 100 nM MitoTracker Red CMXROS (Thermo Fisher Scientific, USA). The cells were incubated with MitoTracker for 15 minutes at 37 °C, and then the medium was exchanged with fresh warm medium, and the cells were further incubated for 5 minutes. The cells were washed twice with cold DPBS and fixed with 4% paraformaldehyde (PFA) in DPBS (pH = 7.0) for 5 min at 37 °C. The cells were then washed three times with cold DPBS for CLSM imaging under Leica SP8 microscope. The red channel showed mitochondria, and the green channel showed fluorescence signals from DBB-Ir-F. The Pearson's coefficients R and overlapping coefficients M1 & M2 were calculated by JACoP plugin in ImageJ.[3] Scatter plots were generated by Colocalization in ImageJ (The particles obviously out of cells were manually excluded from analysis).

3.5. Mitochondrial Membrane Potential Assessment

The depolarization of mitochondrial membrane potential after BP treatment was evaluated by using JC-1 mitochondrial potential sensors (Invitrogen) under CLSM. CT26 cells (2×10^5 cells) were seeded in 35 mm glass bottom dishes and cultured overnight. PBS, Hf_{12}-Ir, BP or BP/Hf_{12}-Ir was added each dish at a Hf concentration of 125 μM or/and a BP concentration of 10 μM. After incubation for 6 hours, JC-1 sensors were added to the dishes at a concentration of 10 μM. After incubation for 30 minutes, the cells were washed twice with PBS and then RPMI-1640 medium was added. The cells were subsequently imaged using a Leica SP8 microscope.

3.6. *In Vitro* ROS Generation

In vitro ROS production of PBS, Hf_{12}-Ir, BP or BP/Hf_{12}-Ir under X-ray irradiation was detected by staining cells with 2',7'-dichlorodihydrofluorescein diacetate (DCFH-DA). CT26 cells were seeded in Petri dishes (1.5×10^5 cells per well) and cultured overnight. The cells were incubated with PBS, Hf_{12}-Ir, BP or BP/Hf_{12}-Ir (125 μM Hf or 5 μM BP) for 8 h, followed by addition of DCFH-DA (40 μM). After incubation for 30 minutes, the cells were irradiated with X-ray (6 Gy), and then stained with Hoechest 33342 for 5 minutes, washed twice with PBS, and imaged by CLSM. ROS generation without X-ray irradiation was measured for comparison.

3.7. In Vitro Efficacy Assays

Cell Viability Assay: The cytotoxicity of Hf_{12}-Ir, BP or BP/Hf_{12}-Ir was assessed by 3-(4,5-dimethylthiazol-2-yl)-5-(3-carboxymethoxyphenyl)-2-(4-sulfophenyl)-2H-tetrazolium (MTS) assay (Promega, Madison, USA) according to the manufacturer's protocol. Briefly, CT26 cells were seeded in 96-well plates (5×10^3 cells per well) and incubated overnight. Hf_{12}-Ir, BP, or BP/Hf_{12}-Ir was added to the cells at different concentrations and incubated for 48 h. The cell viability was determined by MTS assay.

Apoptosis Analysis: The apoptosis of CT26 cells after different treatments was evaluated by flow cytometry. CT26 cells were seeded in 6-well plates (2×10^5 cells per well) and incubated overnight. PBS, Hf_{12}-Ir, BP, or BP/Hf_{12}-Ir was added to the cells at a Hf concentration of 125 μM or/and a BP concentration of 10 μM. After incubation for 24 h, the cells were collected and stained with propidium iodide (PI) and Alexa Fluor 488-Annexin V. The apoptosis rate was analyzed by flow cytometry.

Western Blot: Western blot was performed to evaluate DNA damage caused by X-rays. CT26 cells were seeded in 6-well plates at a density of 2.5×10^5 cells/well and cultured overnight. The cells were incubated with PBS, Hf_{12}-Ir, or BP/Hf_{12}-Ir at a Hf concentration of 125 μM for 8 hours. The cells treated with Hf_{12}-Ir or BP/Hf_{12}-Ir were irradiated with 6 Gy X-ray (n = 3). After 12 h of incubation, the cells were lysed by RIPA buffer with protease inhibitor following the manufacturer's specification. The proteins in the supernatants were collected by centrifugation at 14000 g, and the concentrations were quantified by BCA assay. The proteins were denatured and reduced by NuPAGE™ LDS sample buffer with 50 mM DTT, and then heated to 90 °C for 10 min. Forty μg of samples were loaded on NuPAGE™ 4 to 12%, Bis-Tris gel for electrophoresis on an XCell SureLock™ Mini-Cell (200V, 40 minutes), and electrotransferred to PVDF membrane (300 mA, 120 min) on a mini trans-blot electrophoretic transfer cell. The membrane was blocked by TBST with 5% non-fat dry milk at room temperature for 20 minutes and incubated with primary antibody solution in TBST with 5% BSA at 4°C overnight (Phospho-histone H2A.X (Ser139) (20E3) rabbit mAb #9718, 1:2000; β-Actin (8H10D10) mouse mAb #3700, 1:2000, Cell Signaling Technology, USA). The membrane was washed with TBST and incubated with a secondary antibody with HRP conjugate in TBST with 5% BSA at room temperature for 2 hours (anti-rabbit IgG, HRP-linked antibody #7074, 1:10000; anti-mouse IgG, HRP-linked antibody #7076, 1:10000, Cell Signaling Technology, USA). The membrane was

washed again with TBST, followed by the addition of Pierce™ ECL western blotting substrate. The chemiluminescence was then recorded by a FluorChem R system.

Colony Assay: The colony assay was performed to evaluate the proliferative ability of CT26 cells after different treatments. CT26 cells were seeded in 6-well plates at a density of 1.0×10^5 cells/well and cultured overnight. The cells were incubated with PBS, Hf_{12}-Ir, or BP/Hf_{12}-Ir at a Hf concentration of 125 μM for 8 hours, and then irradiated with 0, 2, 4, 6 and 8 Gy X-ray (n = 3). The cells were washed twice with PBS and trypsinized to obtain single cells. The cells were counted and diluted, and then 200 cells were seeded in each well of 6-well plates and further cultured in 2 mL medium for 7 days. Then, the plates were rinsed once with PBS, fixed by 4% paraformaldehyde for 20 minutes and washed with PBS twice at room temperature. After that, the colonies were manually counted, and the confluence was used as a parameter to calculate the plating efficiency (PE) and surviving fraction (SF):

PE = Confluence (0 Gy, PBS) / Cell seeding number

SF (Dose, Drug) = Confluence (Dose, Drug) / Cell seeding number × PE

3.8. *In Vitro* Metabolism Study

Intracellular Adenosine triphosphate (ATP) and Glutathione (GSH) Measurements: CT26 cells were seeded in 96-wells at a density of 8000 cells/well. PBS, Hf_{12}-Ir, BP, or BP/Hf_{12}-Ir was added into the cells at a Hf concentration of 125 μM or/and a BP concentration of 10 μM. After incubation for 24 h, the cells were lysed with RIPA buffer and centrifuged at 14,000 x g for 5 minutes at 4 °C. The supernatants were collected for intracellular ATP and GSH measurements using the ATP determination kit and GSH determination kit.

Intracellular Hexokinase II (HKII) and Succinate Dehydrogenase (SDH) Activity Measurements: CT26 cells were seeded in 6-well plates at a density of 5×10^5 cells/well and cultured overnight. The cells were incubated with PBS, BP or BP/Hf_{12}-Ir at a Hf concentration of 125 μM or/and a BP concentration of 10 μM. After 24 h of incubation, the cells were harvested and resuspended in HKII assay buffer or SDH assay buffer. The cells were homogenized by freeze-thaw cycles in liquid nitrogen three times, followed by centrifugation at 14,000 x g for 5 minutes at 4 °C. The supernatants were collected for intracellular HKII and SDH activity measurements using the HKII and SDH determination kits, respectively.

Intracellular Glyceraldehyde 3-phosphate dehydrogenase (GAPDH) Measurements: CT26 cells were seeded in 6-well plates at a density of 5 × 10^5 cells/well and cultured overnight. The cells were incubated with PBS, BP or BP/Hf_{12}-Ir at a Hf concentration of 125 μM or/and a BP concentration of 10 μM. After 24 h of incubation, the cells were harvested and washed with cold PBS. The cells were then resuspended in 100 μL of ice cold GAPDH assay buffer and homogenized quickly by pipetting up and down a few times. The cells were incubated on ice for 10 minutes and then centrifuged at 10,000 x g for 5 minutes at 4 °C. The supernatants were collected for intracellular GAPDH activity measurements using the GAPDH determination kit.

Hypoxia Alleviation Assay: The hypoxia alleviation of CT26 cells after BP treatment was evaluated using the hypoxia sensor $Ru(dpp)_3Cl_2$ under CLSM. CT26 cells were seeded on 35 mm glass bottom dishes at a density of 2 × 10^5 cells/dish and cultured in an incubator with 20 % O_2 and 5% CO_2 overnight. After incubation with PBS, Hf_{12}-Ir, BP or BP/Hf_{12}-Ir at a Hf concentration of 125 μM or a BP concentration of 10 μM, the cells were transferred into a hypoxia chamber (0.5 % O_2 and 5% CO_2) for 6 h. $Ru(dpp)_3Cl_2$ was then added to the dishes at a concentration of 10 μM. After 20 min of incubation, the cells were washed twice with PBS, stained with Hoechst 33342, washed twice with PBS. The cells were added with RPMI-1640 medium and then imaged by CLSM.

3.9. *In vivo* Anti-tumor Efficacy Studies

CT26 cells were subcutaneously (*s.c.*) injected into the left flanks of 6-week-old BALB/c mice (2 × 10^6 cells per mouse) on day 0. When the averaged tumors volume reached 100 mm^3 on day 7, the mice were randomized into five groups (n = 5): i) PBS(-), ii) PBS(+), iii) Hf_{12}-Ir(+), iv) BP(+), and v) BP/Hf_{12}-Ir(+). Mice were intratumorally (*i.t.*) injected with PBS, Hf_{12}-Ir, BP or BP/Hf_{12}-Ir at a Hf_{12}-Ir dose of 0.5 μmol or/and a BP dose of 0.2 μmol on day 7 and 9. Eight hours following each *i.t.* injection, the mice were anesthetized with 2.5% (V/V) isoflurane/O_2 and mounted onto an X-Rad 225 irradiator. The CT26 tumors were irradiated with 2 Gy X-ray/fraction for 6 consecutive days starting on day 7 (plus 8 hours). The PBS(-) group did not receive X-ray irradiation and served as control.

The length (L) and width (W) of the tumors as well as mouse body weights were recorded every other day. The tumor volume (V) was calculated using the formula V (mm^3) = L (mm) × W^2 (mm^2) × 0.5. The tumor

growth inhibition rate (TGI) was calculated following the equation: TGI = (mean tumor volume of the PBS group – mean tumor volume of the treatment group) / mean tumor volume of the PBS group × 100%. The mice were euthanized once the tumor volume of the PBS(-) group exceeded 2000 mm^3. At the endpoint of the experiments, the mice were euthanized, and the tumors and major organs were sectioned for hematoxylin-eosin (H&E) staining to evaluate general toxicity.

3.10. *In vivo* Anti-tumor Efficacy of BP/Hf_{12}-Ir in Combination with αPD-L1

CT26 cells were *s.c.* injected into the left flanks of 6-week-old BALB/c mice (2×10^6 cells for each mouse) on day 0. When the averaged tumors volume reached 100 mm^3 on day 7, the mice were randomized into four groups (n = 5): i) PBS(-), ii) αPD-L1(+), iii) BP/Hf_{12}-Ir(+), and iv) BP/Hf_{12}-Ir + αPD-L1(+). The mice were *i.t.* injected with PBS or BP/Hf_{12}-Ir at a dose of 0.5 μmol Hf_{12}-Ir and 0.2 μmol BP on day 7 and 9. For selected groups, the mice were also intraperitoneally injected with 100 μg αPD-L1 on day 7 and 9. Eight hours following each i.t. injection, the mice were anaesthetized with 2.5% (V/V) isoflurane/O_2, and mounted onto an X-Rad 225 irradiator. At the endpoint of the experiment, the mice were euthanized, and the tumors and major organs were sectioned for hematoxylin-eosin (H&E) staining to evaluate the antitumor efficacy and general toxicity, respectively.

3.11. Immune Cell Profiling

CT26 tumor-bearing BALB/c mice (n = 6) were inoculated and treated in the same way as in the in vivo anti-tumor efficacy study. The tumors were harvested on day 14 for immune cell profiling by flow cytometry. Briefly, each harvested tumor was digested with 600 μL of RPMI-1640 medium (containing 10% FBS, 1 mg/mL collagenase I, 250 μg/mL collagenase IV, and 50 μg/mL DNase I) at 37 °C for 45 minutes, followed by the addition of 5 mL complete RPMI-1640 medium. The mixture was gently ground and filtered through sterile cell strainers (40 μm, Corning) to obtain single-cell suspension (~10^7 cells/mL). The cell pellets were collected by centrifugation at 500 g for 5 minutes at 4 °C. 2 mL of ACK buffer (ThermoFisher) was added into the tube to lyse red blood cells. The remaining cells were centrifuged (500 g, 5 min) at 4 °C, washed with ice-cold FACS buffer, and first stained with LIVE/DEAD™ fixable yellow dead cell stain kit (1: 1000 dilution). The cells were then washed with FACS buffer and stained with fluorochrome-conjugated rat anti-mouse antibodies (1:100 dilution) at 4 °C for 45 minutes. The information

for the antibodies, including conjugated dyes, vendors, and clone numbers, is listed as follows: CD45-BV421 (Biolegend, 30-F11), CD11b-FITC (Invitrogen, M1/70), F4/80-PerCP/Cy5.5 (Biolegend, BM8), CD86-APC (Biolegend, GL1), CD206-PE/Cy7 (Biolegend, C068C2), CD3e-PE/eFluor610 (Invitrogen, 145-2C11), CD8-PerPC-eFluor710 (Invitrogen, 53-6.7), CD4-APC-Cy7 (Biolegend, GK1.5). The cells were centrifuged (500 g, 5 min) at 4 °C, washed, and resuspended in FACS buffer for flow cytometry analysis.

3.12. Immunohistochemistry Analysis

To evaluate DNA damage, hypoxia, and tumor proliferation after different treatments, CT26-bearing mice were established and treated in the same way as in the in vivo antitumor efficacy experiment. The mice were euthanized one day after the last X-ray irradiation. The tumors were excised and fixed in 4 % PFA for 24 hours and 70% ethanol for 24 hours. The tissues were embedded in paraffin, sectioned, and stained for H&E, γ-H2AX, Ki67, TUNEL, and CA9 by the Human Tissue Resource Center at the University of Chicago. Briefly, the slides were deparaffinized and rehydrated using xylenes and serial dilutions of ethanol to distilled water. Then the slides were treated with antigen retrieval buffer (Leica Biosystems, AR9640) and heated in a steamer at 97°C for 20 minutes. After washing with tris-buffered saline (TBS), the slides were incubated with primary γ-H2AX antibody (1:400) or primary Ki67 antibody (Thermo Fisher Scientific, Clone# SP6, 1:400) at room temperature for 1 hour in a wet chamber. The slides were washed with TBS and then γ-H2AX and Ki67 slides were incubated with anti-rabbit-polymer (Bond Polymer Refine Detection, Leica Biosystems, DS9800) for 30 minutes at room temperature. The antigen-antibody binding was detected with the 3,3′-Diaminobenzidine (DAB) (DAKO, K3468) system. Tissue sections were then immersed in hematoxylin for counterstaining and covered with cover glasses. The slides were scanned on a CRi Pannoramic SCAN 40× whole slide scanner by Integrated Light Microscopy Core at the University of Chicago. The images were analyzed with QuPath-0.2.3 and Image J software.

3.13. IFN-γ ELISPOT assay

A Multiscreen HTS-IP plate (Millipore Sigma) was activated with 70% ethanol, washed with DPBS, coated with anti-mouse IFN-γ capture antibody (BD Biosciences) at 37 °C for 2 hours, and blocked with sterile RPMI-1640 medium with fetal bovine serum (FBS, 10%) at room temperature for 2 hours. The spleens

were harvested from the treated CT26 tumor-bearing BALB/C mice and gently ground and filtered through sterile cell strainers to afford single-cell suspensions. Red blood cells were then lysed with sterile ACK buffer (gibco), and splenocytes were counted and seeded in the plate at a density of 2×10^5 cells/well in RPMI-1640 full medium. CT26 tumor-associated SPSYVYHQF (AH1) peptide was added to each well at a concentration of 10 μg/mL. The splenocytes were incubated at 37 °C for 72 hours and the culture media were discarded. The plates were then washed and incubated with biotinylated anti-IFN-γ detection antibody, streptavidin-HRP conjugate, and AEC substrate following the manufacturer's specification (BD Biosciences). The plate was air-dried and analyzed with a CTL ImmunoSpot® S6 Analyzer.

3.14. *In Vivo* Anti-Metastatic Efficacy on 4T1 Tumor Model

The orthotopic 4T1 tumor model was established in BALB/c mice by inoculating 4T1 cells into the mammary pad of 6-week-old BALB/c mice (2×10^6 cells per mouse) on day 0. When the average tumor volume reached 100 mm^3 on day 7, the mice were randomized into four groups (n = 5): i) PBS(-), ii) αPD-L1(+), iii) BP/Hf_{12}-Ir(+), and iv) BP/Hf_{12}-Ir + αPD-L1(+). The mice were *i.t.* injected with corresponding treatments at a dose of 0.5 μmol Hf_{12}-Ir, 0.2 μmol BP, or 100 μg αPD-L1) on day 7 and 9. Eight hours following each *i.t.* injection, the mice were anesthetized with 2.5% (V/V) isoflurane/O_2, and mounted onto an X-Rad 225 irradiator. The 4T1 tumors were irradiated with 2 Gy X-ray/fraction for 6 consecutive days starting on day 7 (plus 8 hours). At the endpoint of the experiment, the mice were euthanized, and the tumors and lungs were sectioned for H&E staining to evaluate their antitumor and antimetastatic effects, respectively.

4. Supplementary Figures

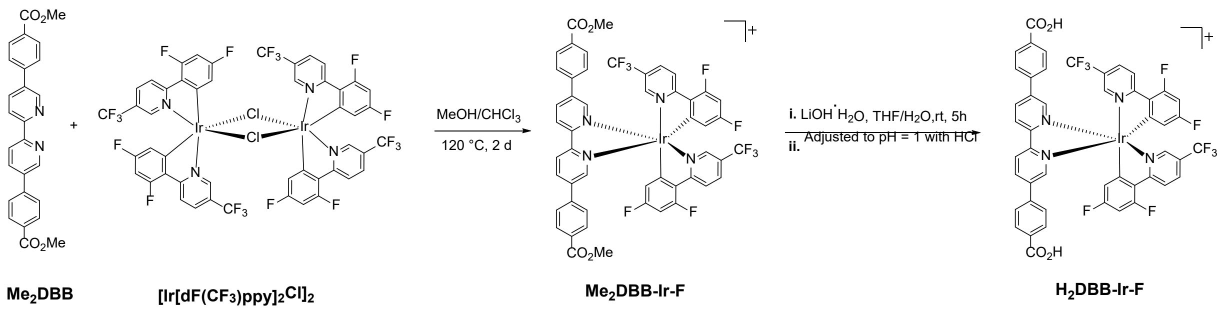

Figure S1. Synthetic route for H_2DBB-Ir-F.

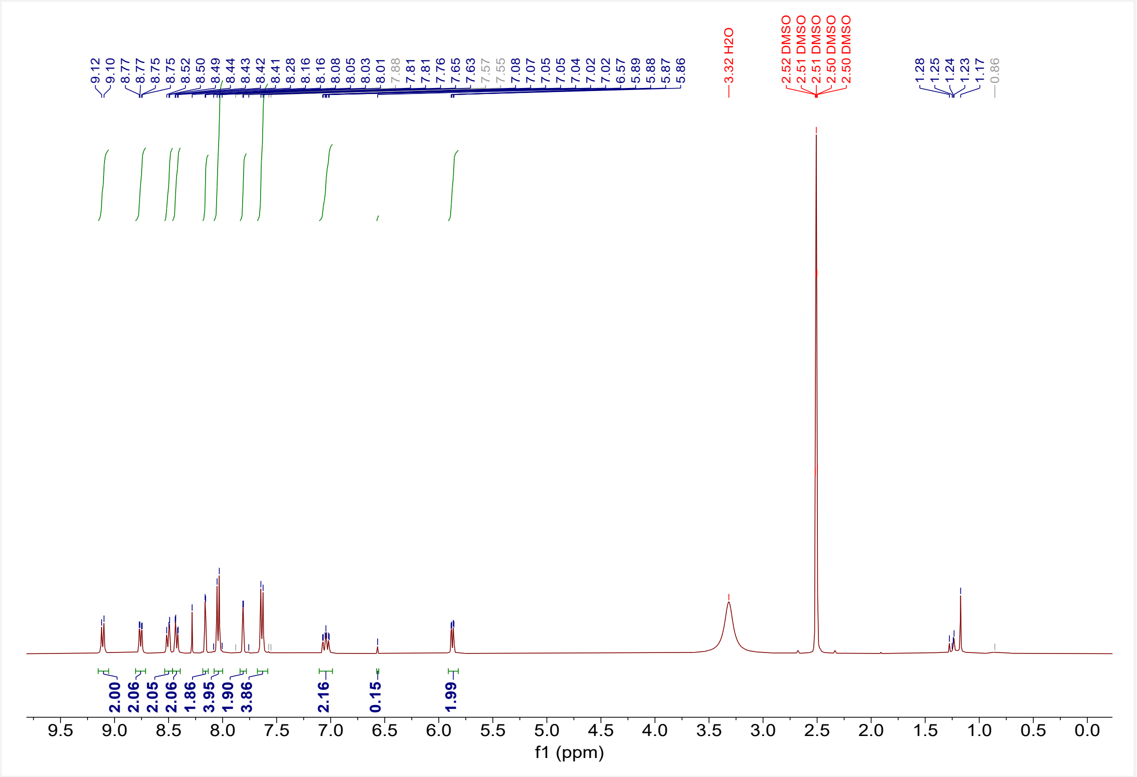

Figure S2. ^{1}H NMR spectrum of H_2DBB-Ir-F in DMSO-d_6.

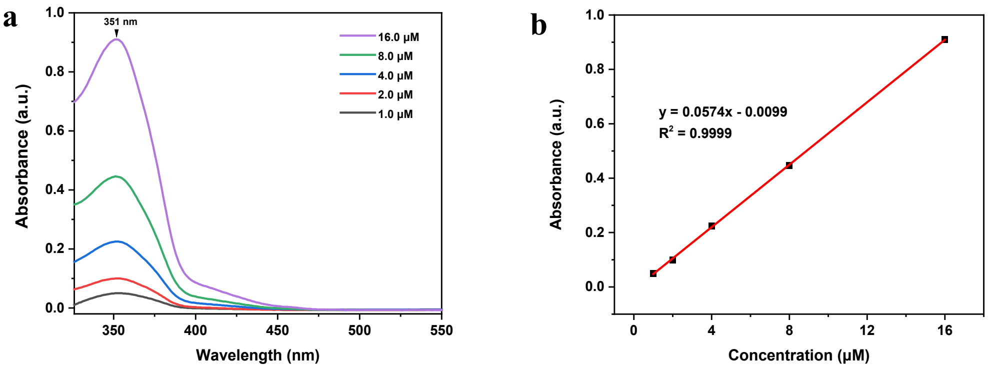

Figure S3. UV-Vis standard curve for H_2DBB-Ir-F. (a) UV-Vis absorption spectra of H_2DBB-Ir-F in DMSO at different concentrations. The characteristic absorption of H_2DBB-Ir-F at 351 nm corresponds to the metal-to-ligand charge-transfer (MLCT) transition. (b) Linear fit of the H_2DBB-Ir-F absorbance at 351 nm as a function of concentration.

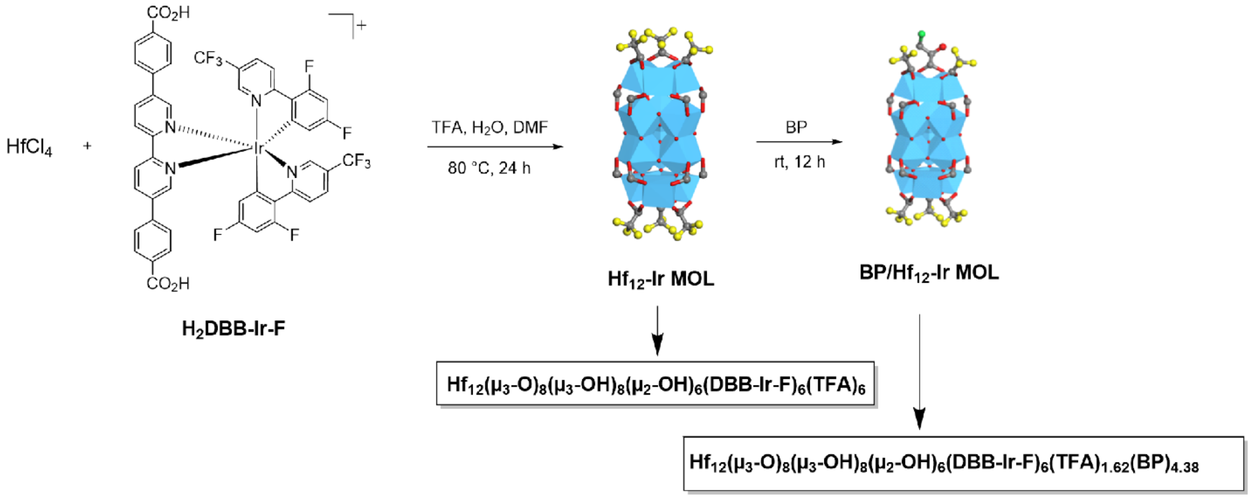

Figure S4. Synthetic route for Hf_{12}-Ir and BP/Hf_{12}-Ir MOLs.

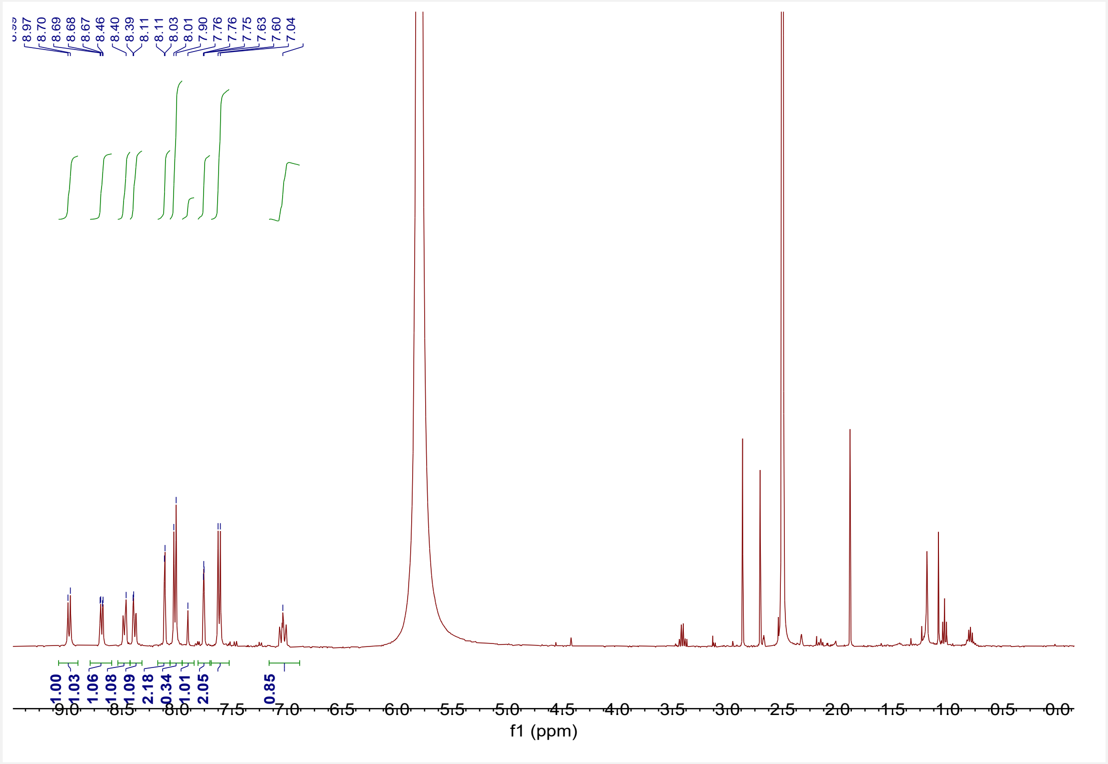

Figure S5. ^{1}H NMR spectrum of digested Hf_{12}-Ir MOL in DMSO-d_6.

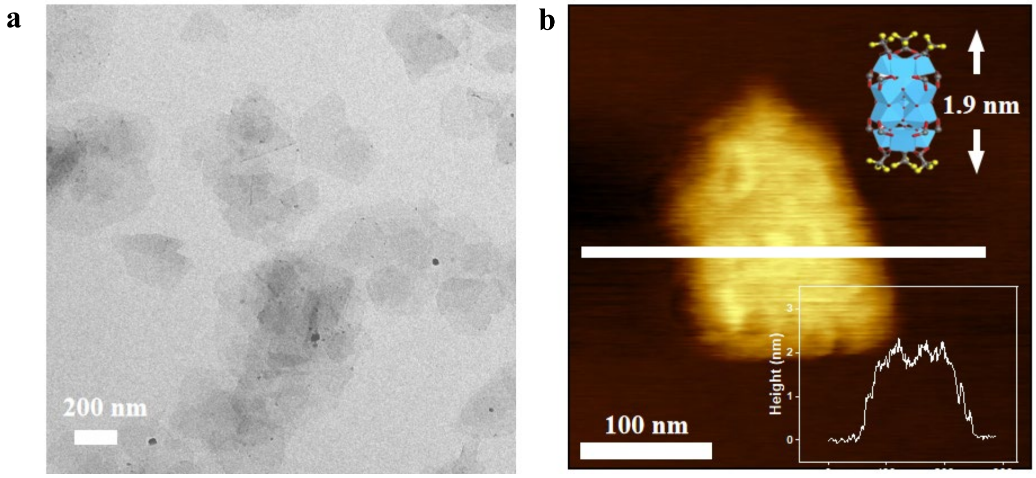

Figure S6. Morphological characterization of Hf_{12}-Ir MOL. (a) TEM image of Hf_{12}-Ir MOL. (b) AFM topography of Hf_{12}-Ir MOL; the inset shows the height profile.

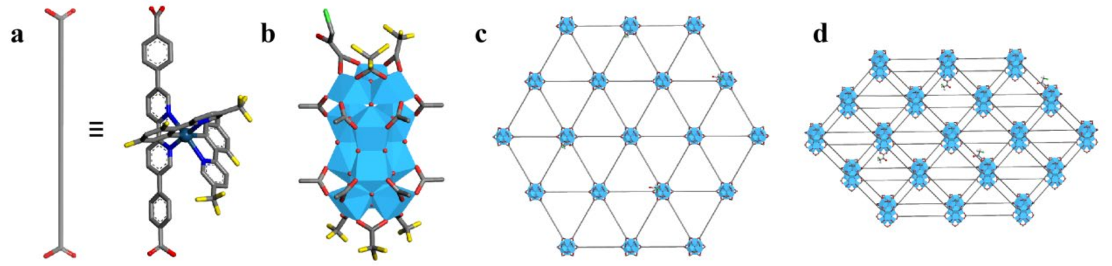

Figure S7. Structure model of BP/Hf_{12}-Ir MOL. (a) Simplified (left) and full (right) structure of DBB-Ir-F bridging ligand. (b) The SBU structure in $Hf_{12}(\mu_3\text{-}O)_8(\mu_3\text{-}OH)_8(\mu_2\text{-}OH)_6(\text{DBB-Ir-F})_6(\text{TFA})_{1.6}(\text{BP})_{4.4}$. (c) A view along the c axis of the BP/Hf_{12}-Ir MOL showing its monolayer morphology. (d) Side view of the BP/Hf_{12}-Ir MOL showing the positions of the bridging DBB-Ir-F ligands in the monolayered framework (dark blue: Ir, sky blue: Hf, yellow: F, green: Br, red: O, grey: C; H atoms are omitted for clarity).

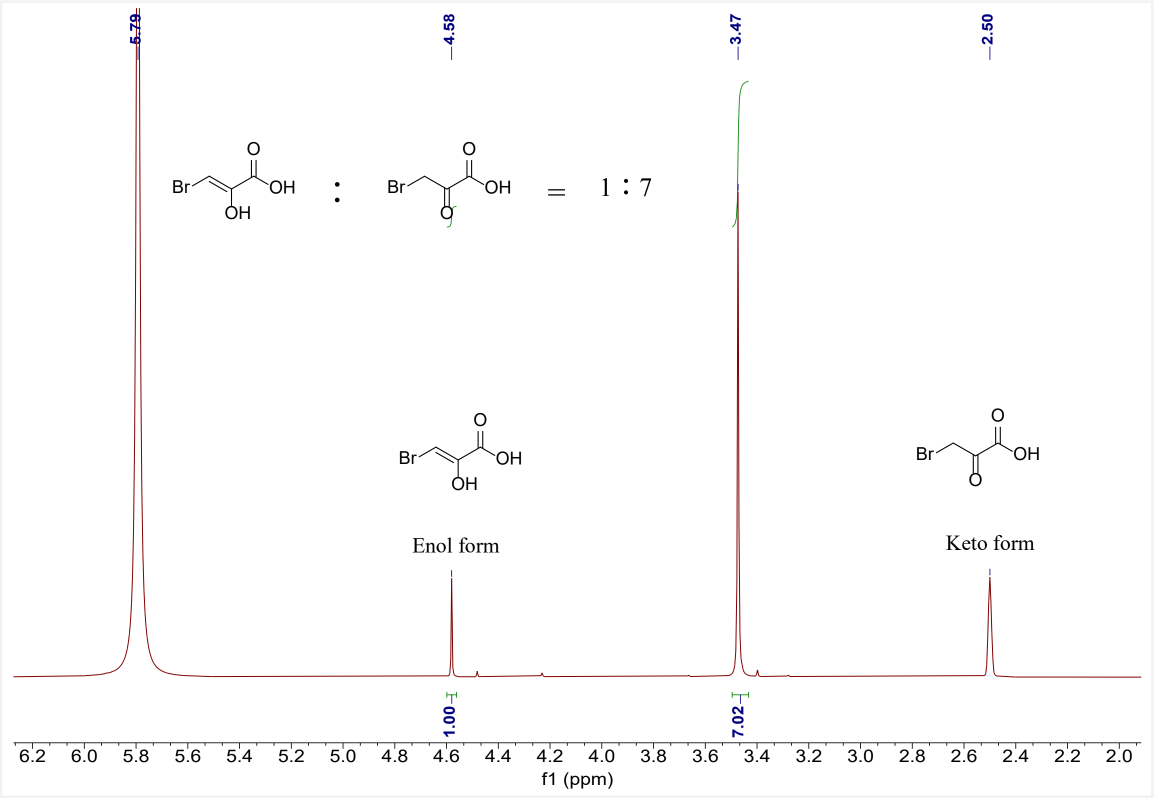

Figure S8. ^1H NMR spectrum of BP in DMSO-d_6.

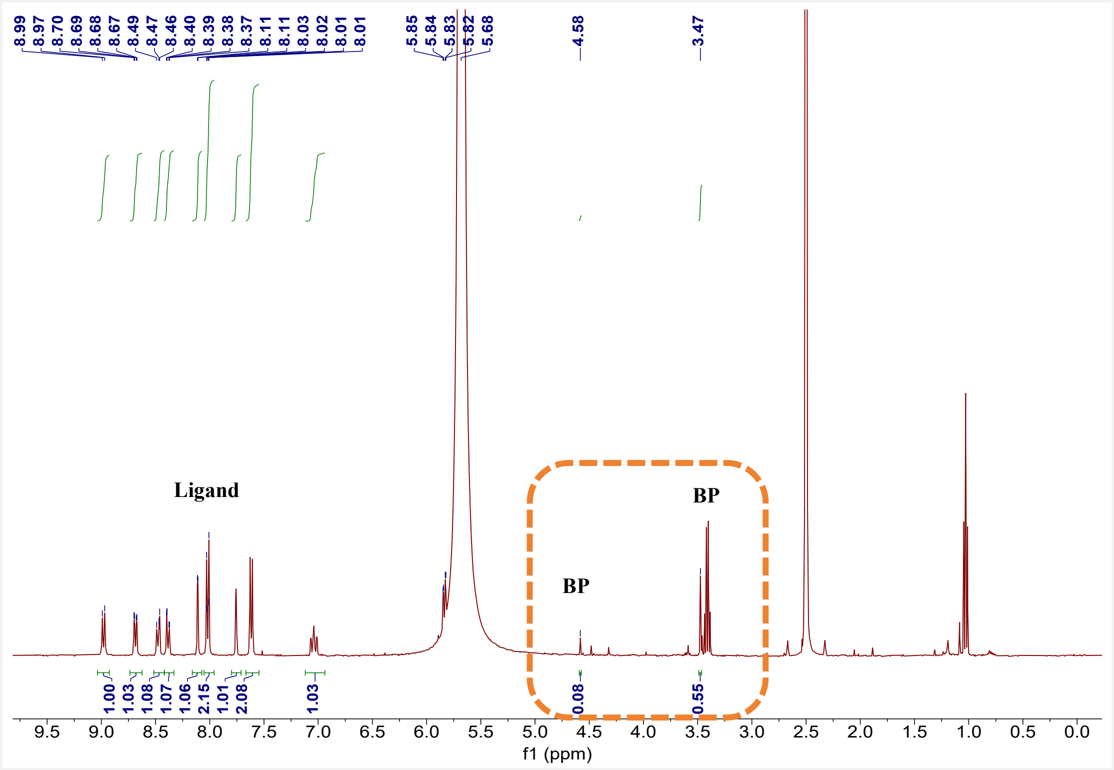

Figure S9. ^{1}H NMR spectrum of digested BP/Hf_{12}-Ir in DMSO-d_6.

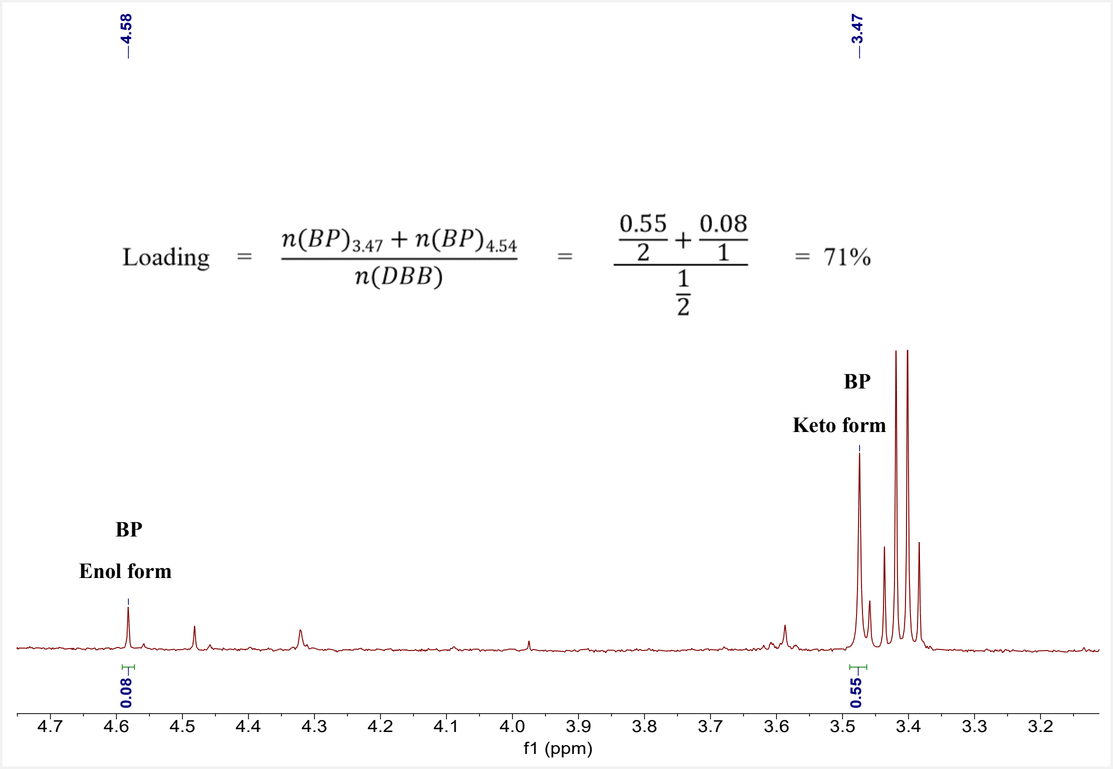

$$\text{Loading} = \frac{n(BP)_{3.47} + n(BP)_{4.54}}{n(DBB)} = \frac{\frac{0.55}{2} + \frac{0.08}{1}}{\frac{1}{2}} = 71\%$$

Figure S10. Zoomed-in view of ^{1}H NMR spectrum of digested BP/Hf_{12}-Ir and the calculated BP loading.

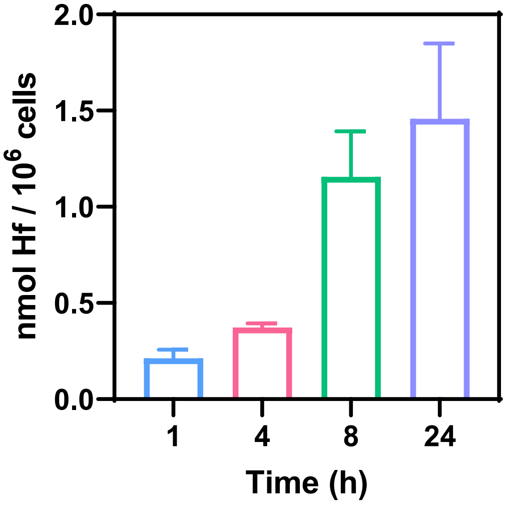

Figure S11. Time-dependent cellular uptake of BP/Hf_{12}-Ir (n = 3).

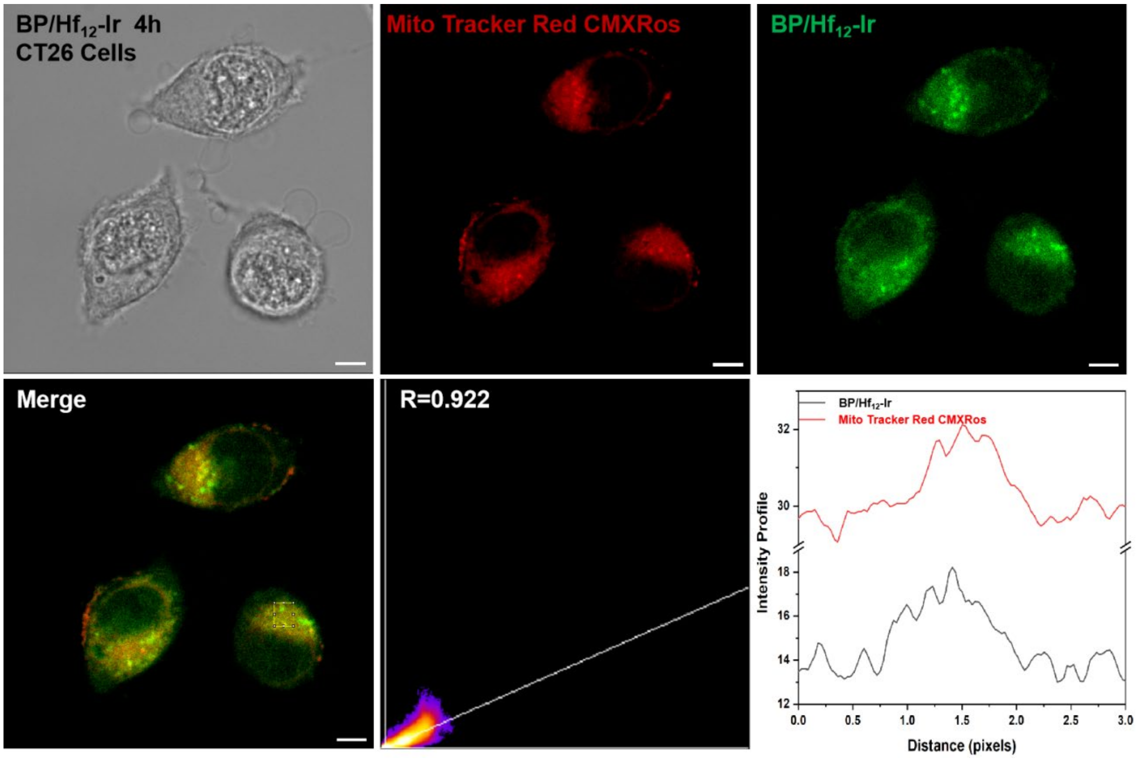

Figure S12. CLSM images and colocalization of BP/Hf_{12}-Ir with mitochondria in CT26 cells; Pearson's R value and scatter plots were generated by Colocalization in ImageJ.

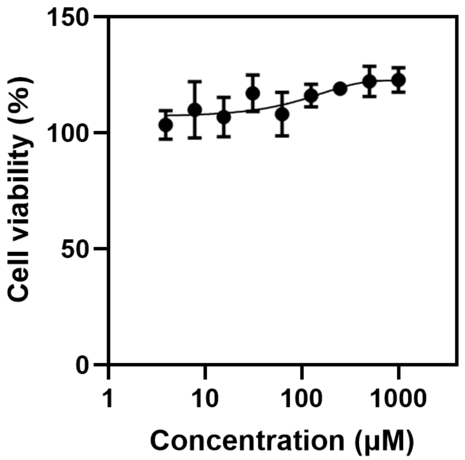

Figure S13. Viability of CT26 cancer cells after treatment with different concentrations of Hf_{12}-Ir MOL.

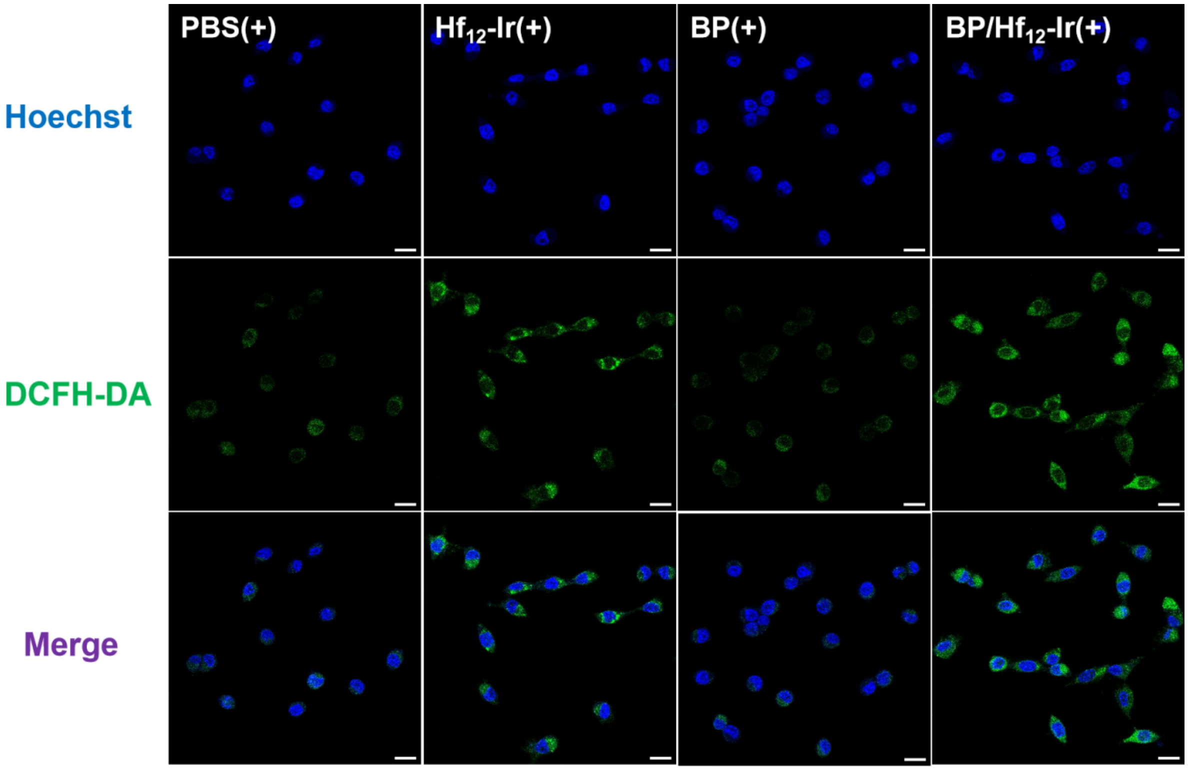

Figure S14. CLSM of total ROS generation in CT26 cells after different treatments with X-ray irradiation (DCFH-DA, green; Hoechst, blue; scale bar = 20 μm).

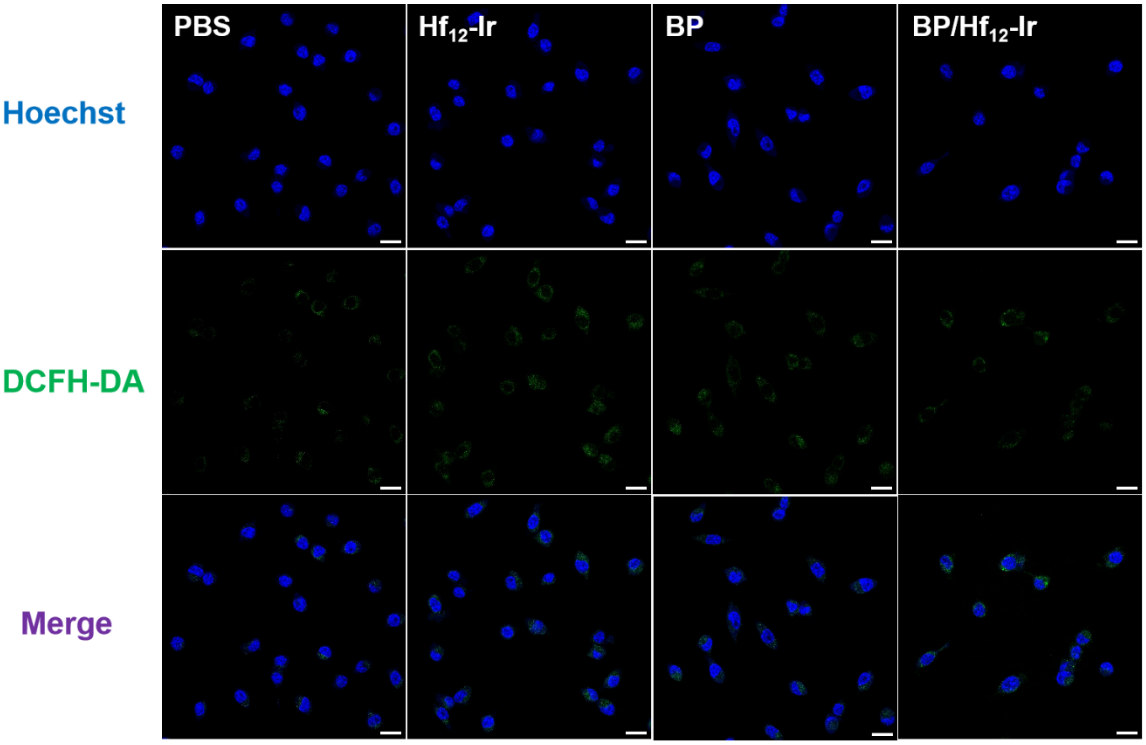

Figure S15. CLSM of total ROS generation in CT26 cells after different treatments without X-ray irradiation (DCFH-DA, green; Hoechst, blue; scale bar = 20 μm).

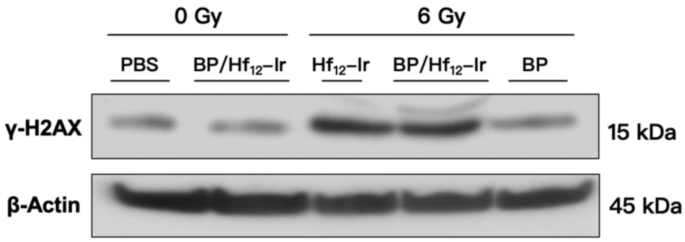

Figure S16. Western blot analysis of DNA double-strand breaks (DSBs) quantified at 12 h after irradiation of CT26 cells with X-ray (6 Gy).

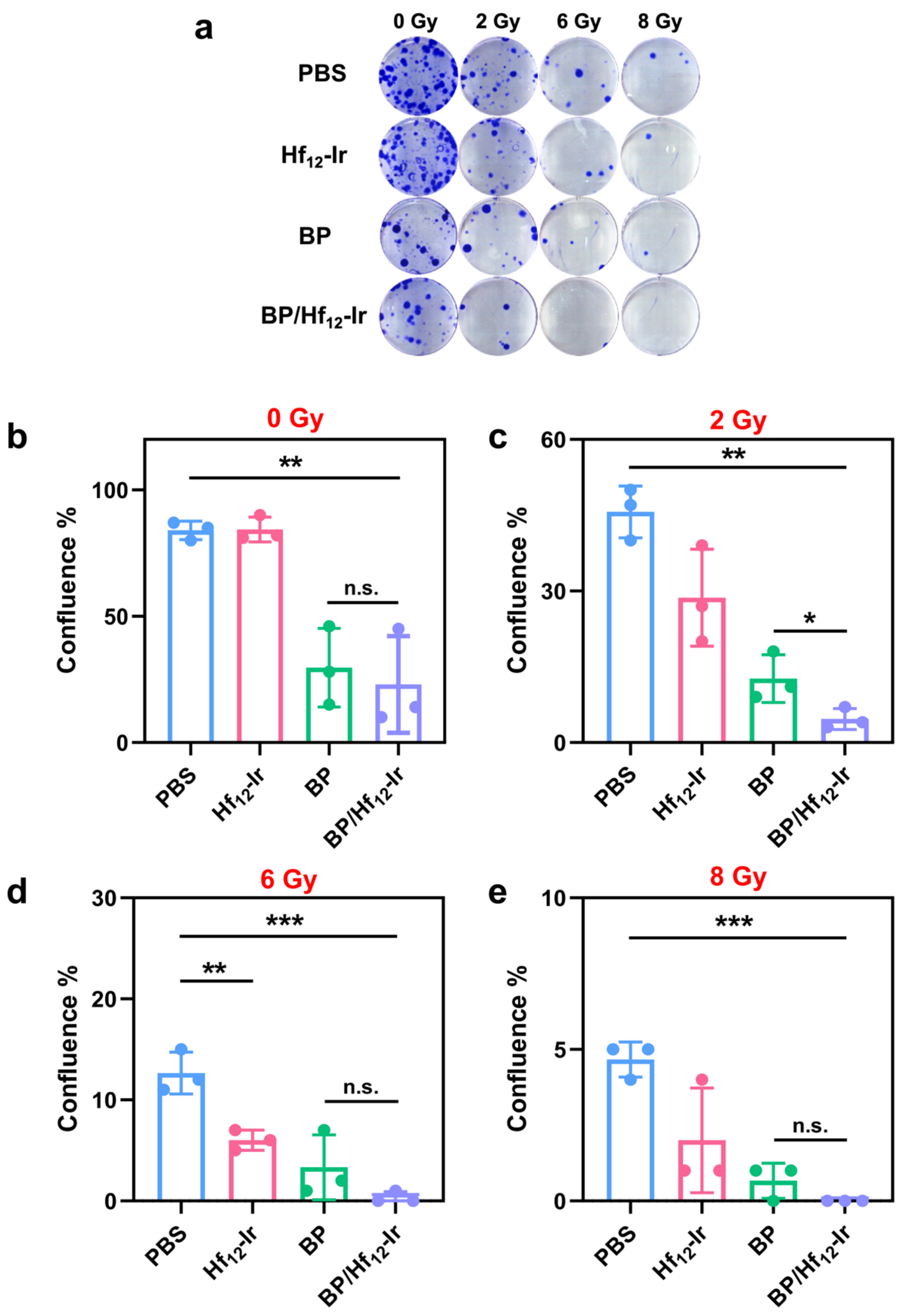

Figure S17. Colony forming assay showing effect of BP combining RT at different RT dose (0, 2, 6, and 8 Gy) on the colony forming abilities (a) and survival fraction (b) (n = 3).

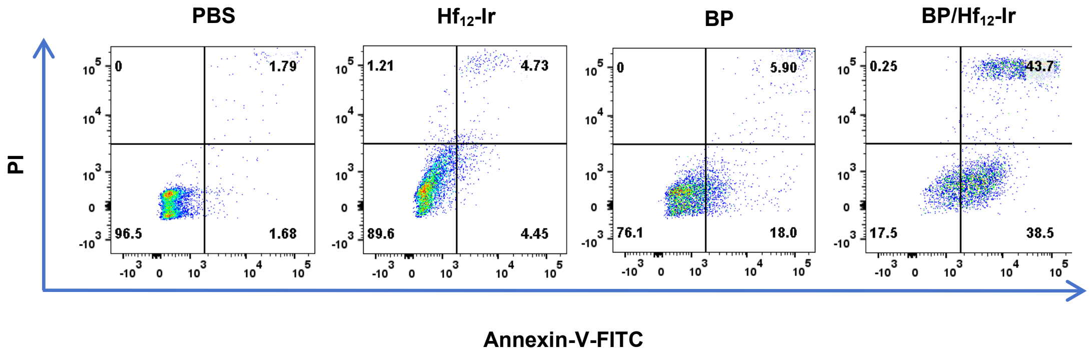

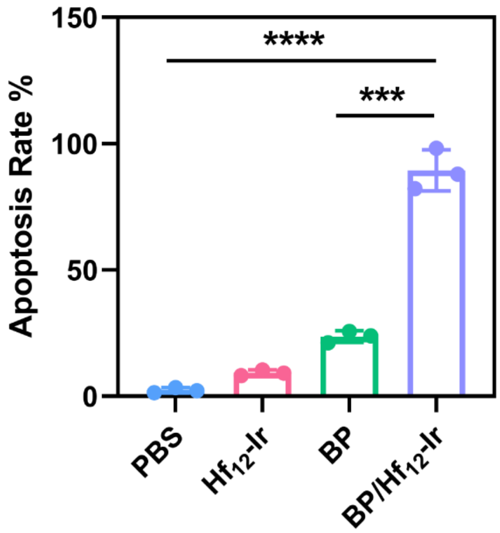

Figure S18. Representative flow cytometric plots and quantification of apoptotic CT26 cells after indicated treatments without X-ray (n = 3).

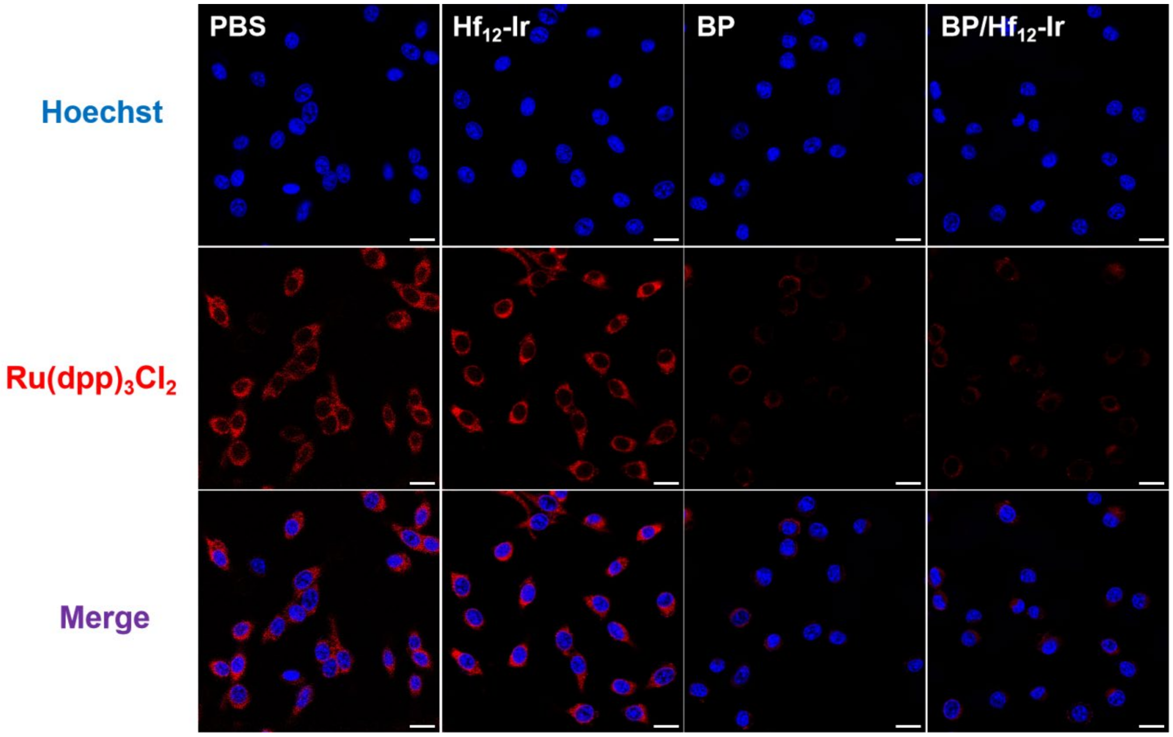

Figure S19. CLSM imaging of $Ru(dpp)_3Cl_2$ in CT26 cells after different treatments.

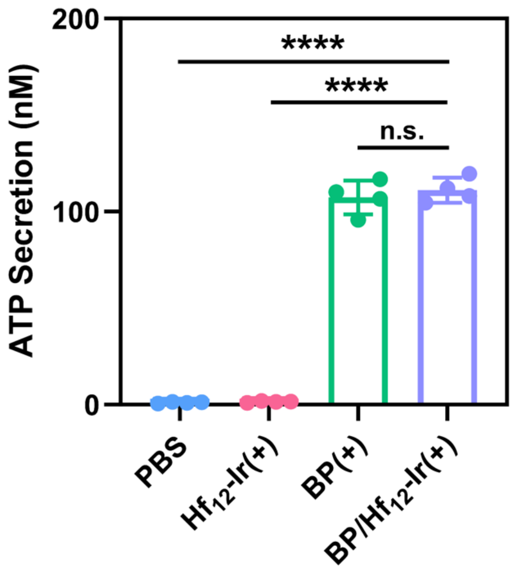

Figure S20. ATP secretion from CT26 cells after different treatments by luciferase conversion assay (n = 3).

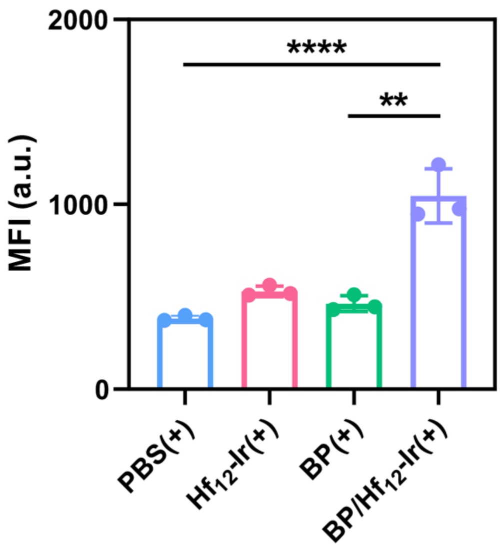

Figure S21. Flow cytometric analysis of CRT expression in CT26 cells 24 hours after different treatments (n = 3).

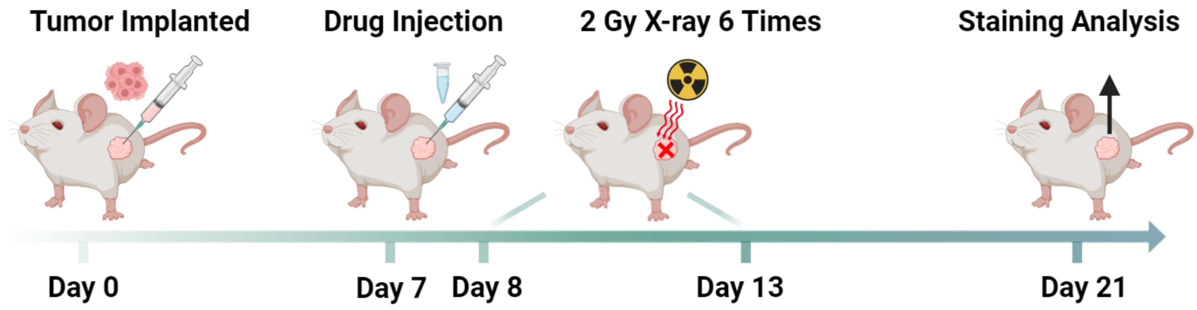

Figure S22. Schematic illustration of tumor inoculation and treatment schedules.

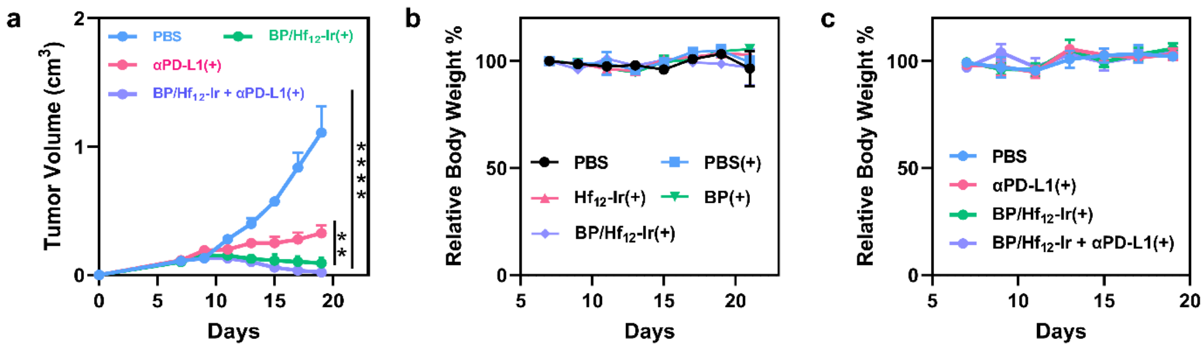

Figure S23. (a) Growth curves, (b) and (c) Relative body weight curves of CT26 subcutaneous tumors after different treatments, n = 5.

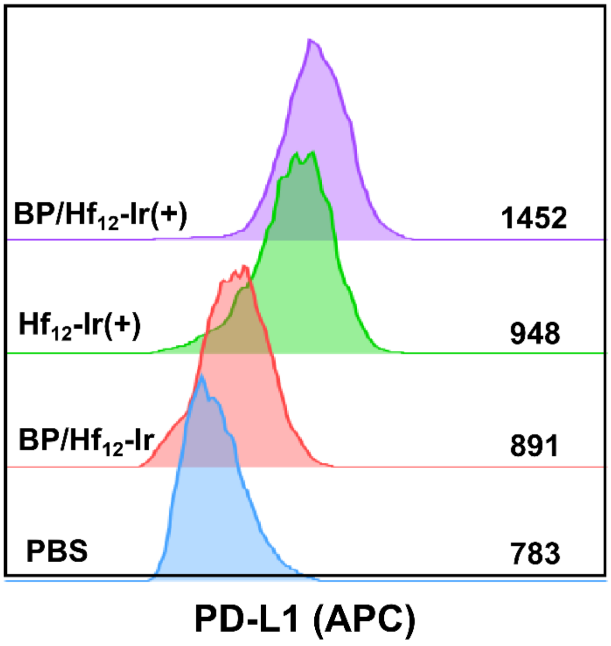

Figure S24. PD-L1 expression in CT26 cells after different treatments.

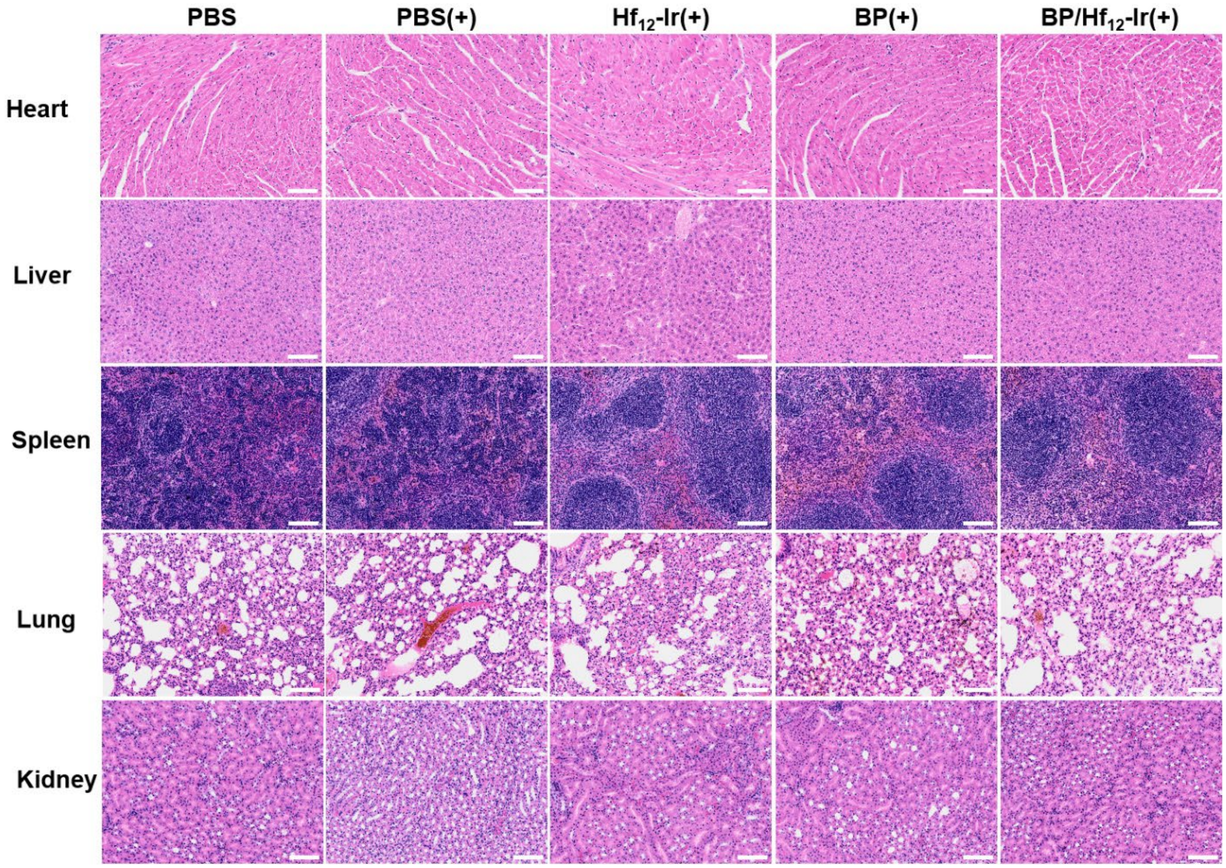

Figure S25. H&E staining of major organs from subcutaneous CT26 tumor-bearing mice after different treatments. Scale bars = 50 μm.

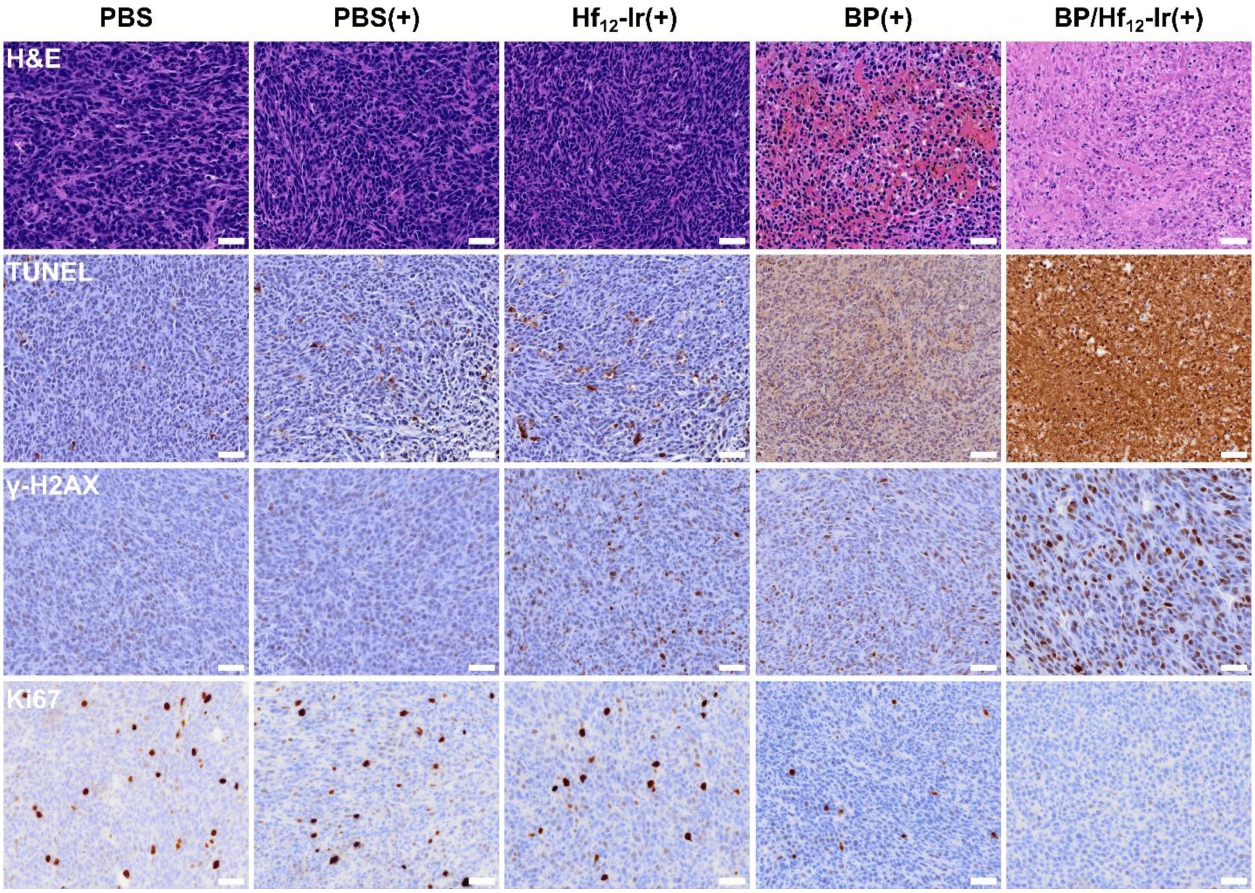

Figure S26. H&E, TUNEL, γ-H2AX and Ki67 staining of CT26 tumors after different treatments. Scale bars = 40 μm.

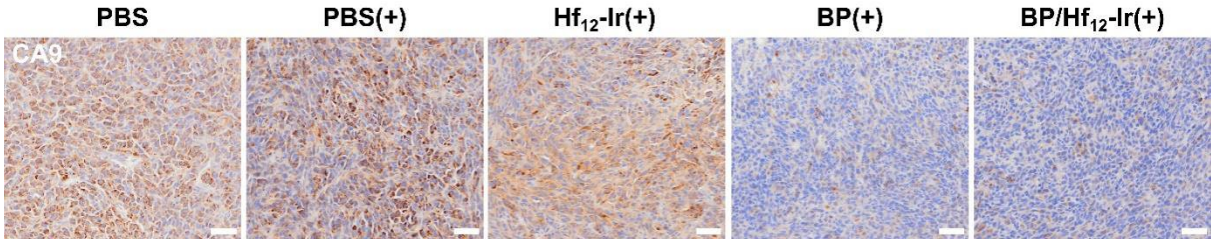

Figure S27.CA9 staining for CT26 tumors hypoxia after different treatments. Scale bars = 40 μm.

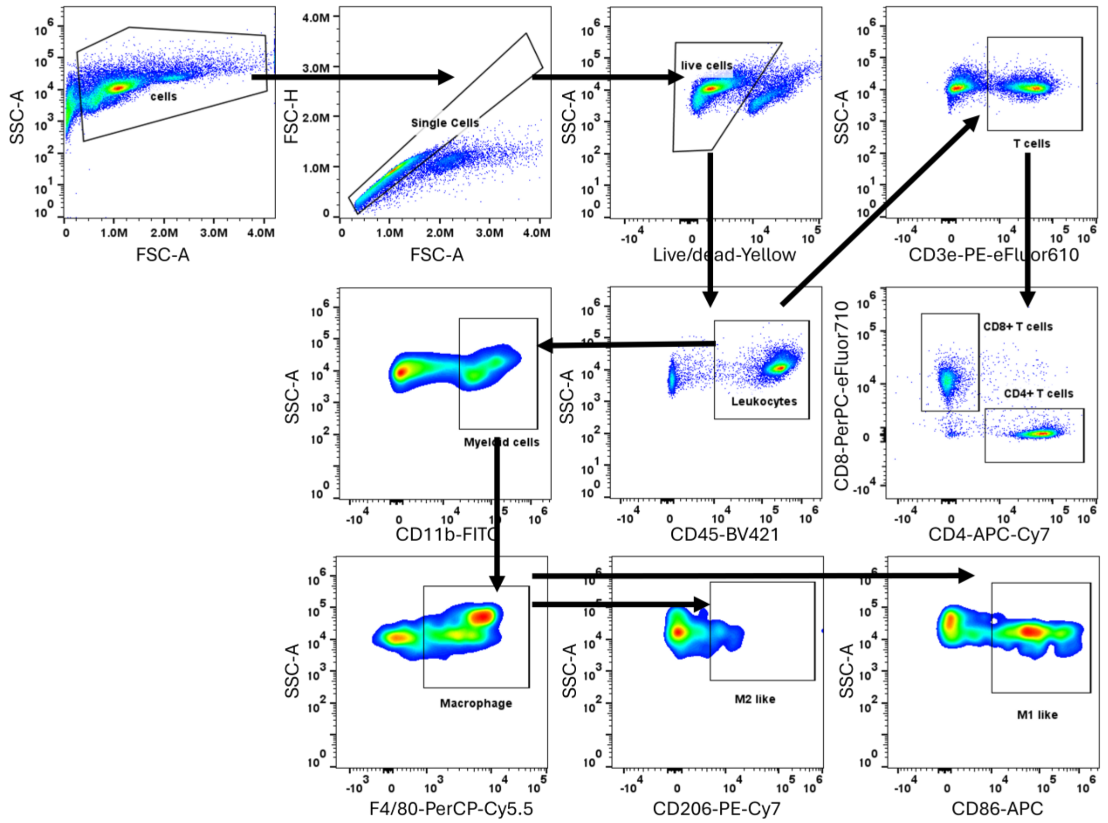

Figure S28. Gating strategies for T cells (CD45⁺ CD3e⁺), cytotoxic T cells (CD45⁺ CD3e⁺ CD8⁺), helper T cells (CD45⁺ CD3e⁺ CD4⁺), and M1 macrophages (CD45⁺ CD11b⁺ F4/80⁺ CD86⁺), and M2 macrophages (CD45⁺ CD11b⁺ F4/80⁺ CD206⁺) in Figure 5.

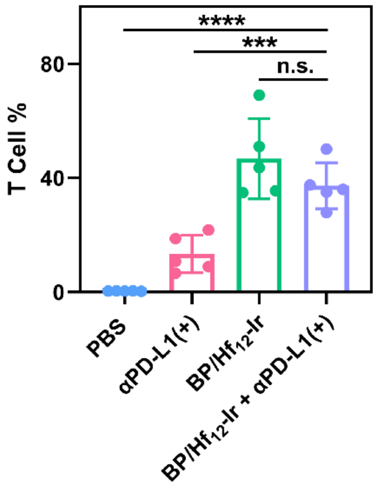

Figure S29. The quantification of T cells, in the tumors from CT26 tumor-bearing mice (n = 5).

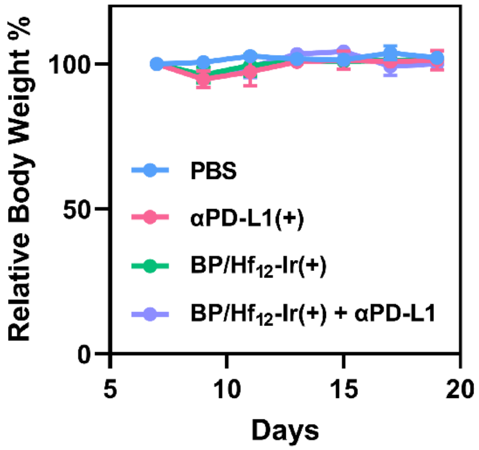

Figure S30. Relative body weight curves of orthotopic 4T1 tumor-bearing BALB/c mice after indicated treatments (n = 5).

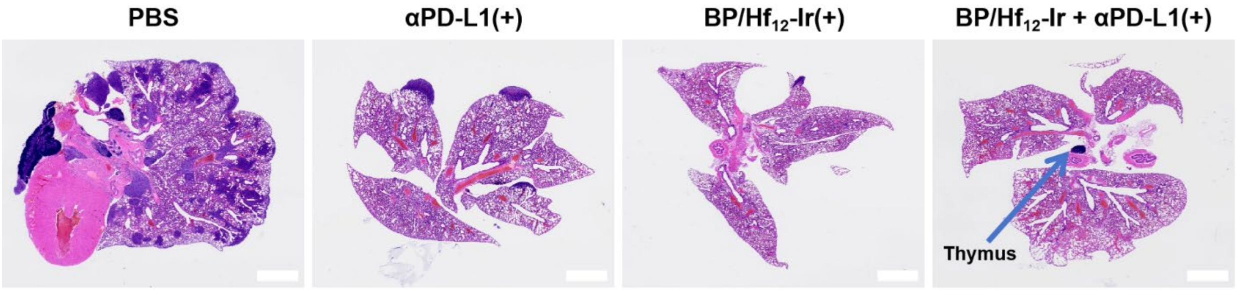

Figure 31. H&E staining for lung metastasis after indicated treatments. (scale bar = 2 mm).

Table S1. The lung metastasis after indicated treatments.

	Tumor cell number	Lung cell number	Metastatic rate (%)
PBS	252656	970463	26.0
α PD-L1(+)	19247	302915	6.4
BP/Hf_{12}-Ir(+)	2161	138658	1.6
BP/Hf_{12}-Ir +αPD-L1(+)	0	288629	0

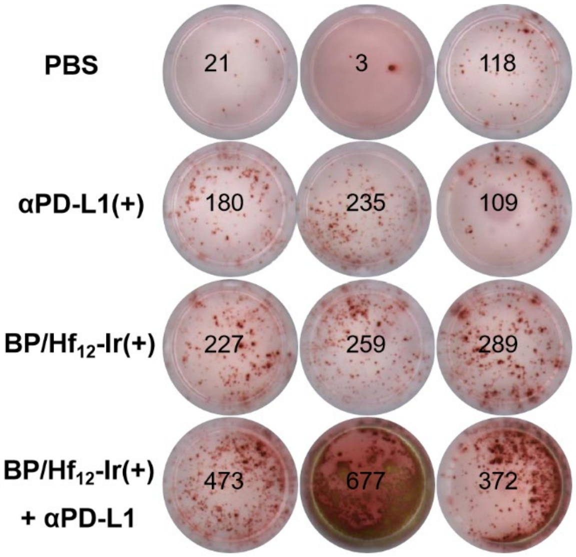

Figure S32. The photos and analysis of the ELISPOT assay detecting CT26 tumor-specific IFN-γ secreting splenocytes (n=3).

Table S2. Tumor volumes of CT26 model (mm^3)

Time(day)	PBS (-)					PBS (+)					Hf12-Ir (+)					BP (+)					BP/Hf12-Ir (+)				
0	0.00	0.00	0.00	0.00	0.00	0.00	0.00	0.00	0.00	0.00	0.00	0.00	0.00	0.00	0.00	0.00	0.00	0.00	0.00	0.00	0.00	0.00	0.00	0.00	0.00
7	67.50	96.23	115.34	126.51	140.40	68.80	169.00	125.09	125.44	89.89	59.74	107.39	134.02	145.12	84.56	63.88	83.75	108.00	112.36	133.96	84.70	117.60	84.27	78.75	147.99
9	97.63	134.54	184.32	143.75	149.96	113.75	196.00	139.24	119.17	121.09	129.60	110.81	179.56	146.33	115.20	82.84	116.03	113.49	150.70	132.28	181.48	128.56	142.23	89.38	231.81
11	249.14	241.06	377.60	279.86	249.90	186.23	305.34	136.13	240.43	157.22	139.24	139.61	181.68	186.05	172.83	133.21	128.80	169.00	194.56	186.29	164.25	120.33	142.88	146.33	188.54
13	361.00	427.43	558.62	329.12	323.44	192.60	315.17	130.14	227.85	178.96	158.44	98.84	202.01	242.76	204.16	167.45	130.54	205.80	199.96	205.80	125.02	111.33	110.47	103.97	188.01
15	400.80	640.00	596.23	550.16	495.14	349.83	534.58	133.10	305.34	219.49	204.72	142.20	177.45	264.24	248.37	139.15	120.74	166.46	269.69	234.41	137.22	69.70	60.75	111.01	184.32
17	498.68	1022.66	1000.69	1047.21	622.26	350.46	472.02	231.18	335.01	237.28	193.55	145.80	219.01	245.00	261.61	155.95	130.14	149.50	303.24	402.69	128.77	98.32	53.62	79.33	160.74
19	593.06	1675.66	1200.56	1356.24	816.75	484.00	569.92	198.66	514.62	318.03	146.07	160.74	219.28	200.69	317.52	217.72	126.71	90.40	355.20	438.89	119.07	75.63	50.85	63.36	157.70
21	741.58	2114.02	1664.65	1918.44	1342.81	530.55	536.99	160.20	795.71	410.50	132.10	122.30	194.21	198.58	335.96	222.21	120.74	117.21	376.54	513.81	99.24	57.13	40.80	47.59	127.83

Table S3. Tumor volumes of 4T1 model (mm^3)

Time(day)	PBS (-)					αPD-L1 (+)					BP/Hf12-Ir (+)					BP/Hf12-Ir(+) + αPD-L1				
0	0.00	0.00	0.00	0.00	0.00	0.00	0.00	0.00	0.00	0.00	0.00	0.00	0.00	0.00	0.00	0.00	0.00	0.00	0.00	0.00
7	78.68	99.14	86.53	70.01	80.12	99.83	70.00	83.82	88.94	71.79	57.43	59.17	72.90	154.79	113.13	84.00	88.00	78.75	80.75	93.78
9	137.92	168.78	142.23	109.80	195.20	147.88	112.09	126.51	140.78	118.35	102.85	62.92	35.64	261.61	152.63	164.78	96.33	102.34	107.65	146.85
11	184.05	296.23	158.76	132.62	213.55	169.88	136.46	146.85	150.72	118.59	90.60	56.14	31.85	200.90	99.24	117.00	53.08	54.68	94.22	135.00
13	201.14	361.25	240.12	233.44	230.63	196.00	153.78	171.09	194.21	206.49	82.34	57.65	36.10	117.74	140.90	109.80	25.11	67.37	84.56	154.64
15	479.86	522.45	377.20	356.33	292.49	209.20	163.87	135.82	181.3	158.76	60.90	56.14	34.46	107.65	124.93	80.06	0.00	50.00	81.65	115.35
17	724.72	1056.45	523.42	567.58	406.50	236.82	161.60	157.12	181.68	233.28	51.23	58.48	0.00	87.48	108.00	65.84	0.00	48.60	41.07	77.06
19	1239.30	1479.80	921.60	810.83	695.75	275.68	219.49	182.65	219.04	198.45	57.22	59.14	0.00	85.76	98.78	0.00	0.00	45.62	0.00	62.50

5. References

1. Y, Quan.; G, Lan.; Y, Fan.; W, Shi.; E, You.; W, Lin., Metal–Organic Layers for Synergistic Lewis Acid and Photoredox Catalysis. *Journal of the American Chemical Society.* **2020,** *142* (3), 1746-1751.

2. Liu, H.; Zhang, Q.; Huang, H.; Qiu, M.; Hu, L.; Zhao, X.; Xu, C.; Wang, Z.; Zhang, C.; Zhan, Y.; Sun, Y. *Biomedical Chromatography.* **2022**, *36 (12)*, 5477.

3. Bolte, S.; Cordelières, F. P., A guided tour into subcellular colocalization analysis in light microscopy. *J. Microsc.* **2006**, *224 (3)*, 213-232.
